# Innate Immunity to Spiral Ganglion Neuron Loss: A Neuroprotective Role of Fractalkine Signaling in Injured Cochlea

**DOI:** 10.3389/fncel.2021.694292

**Published:** 2021-08-02

**Authors:** Andrew Rigel Stothert, Tejbeer Kaur

**Affiliations:** Department of Biomedical Sciences, School of Medicine, Creighton University, Omaha, NE, United States

**Keywords:** sensorineural hearing loss, macrophages, fractalkine, CX_3_CR1, spiral ganglion neurons, neuroprotection, ribbon synapses

## Abstract

Immune system dysregulation is increasingly being attributed to the development of a multitude of neurodegenerative diseases. This, in large part, is due to the delicate relationship that exists between neurons in the central nervous system (CNS) and peripheral nervous system (PNS), and the resident immune cells that aid in homeostasis and immune surveillance within a tissue. Classically, the inner ear was thought to be immune privileged due to the presence of a blood-labyrinth barrier. However, it is now well-established that both vestibular and auditory end organs in the inner ear contain a resident (local) population of macrophages which are the phagocytic cells of the innate-immune system. Upon cochlear sterile injury or infection, there is robust activation of these resident macrophages and a predominant increase in the numbers of macrophages as well as other types of leukocytes. Despite this, the source, nature, fate, and functions of these immune cells during cochlear physiology and pathology remains unclear. Migration of local macrophages and infiltration of bone-marrow-derived peripheral blood macrophages into the damaged cochlea occur through various signaling cascades, mediated by the release of specific chemical signals from damaged sensory and non-sensory cells of the cochlea. One such signaling pathway is CX_3_CL1-CX_3_CR1, or fractalkine (FKN) signaling, a direct line of communication between macrophages and sensory inner hair cells (IHCs) and spiral ganglion neurons (SGNs) of the cochlea. Despite the known importance of this neuron-immune axis in CNS function and pathology, until recently it was not clear whether this signaling axis played a role in macrophage chemotaxis and SGN survival following cochlear injury. In this review, we will explore the importance of innate immunity in neurodegenerative disease development, specifically focusing on the regulation of the CX_3_CL1-CX_3_CR1 axis, and present evidence for a role of FKN signaling in cochlear neuroprotection.

## Introduction

The immune system is the body’s defense mechanism against pathogens and tissue injury. The immune system consists of an intricate network of cells and molecules that work together to protect the body from damage. These cells include lymphoid cells, such as T-cells, B-cells, and NK cells, and myeloid cells, such as monocytes, dendritic cells, macrophages, and granulocytes. Most immune cells, including T/B-cells, monocytes, and macrophages can be found under physiological conditions circulating in the blood. These cells have surface receptors that act in surveillance of the body searching for chemical signals that are released from damaged cells and tissues (Chaplin, 2010). These “find me” signals work to activate immune processes and recruit additional immune cells to the site of injury to initiate the inflammatory response, including phagocytosis (Ravichandran, 2010; Elliott et al., 2017). Many somatic tissues throughout the body have a resident population of macrophages that establish in the tissue embryonically originating from the yolk sac and fetal liver, playing a pivotal role in immune surveillance, tissue development, homeostasis, physiology, and response to injury (Gomez Perdiguero et al., 2015). Examples of these resident macrophages include Langerhans cells (skin), Alveolar macrophages (lungs), Kupffer cells (liver), and microglia [central nervous system (CNS) and peripheral nervous system (PNS)], among many others (Hashimoto et al., 2013; Frodermann and Nahrendorf, 2018; Xie et al., 2019).

Classically, the study of neurodegenerative diseases has focused on the pathological progression of the disease, specifically targeting toxic protein aggregation leading to neuronal dysfunction and death, rather than the underlying disease mechanism (Aguzzi and O’Connor, 2010). Alzheimer’s disease (AD), Parkinson’s disease (PD), Multiple sclerosis (MS), and many other neurodegenerative disorders of the CNS are hallmarked by disruption of memory, sensory, and motor functions due to widespread loss of neurons and their vital communication networks. However, over the past couple of decades, more focus has been centered on the role of neuroinflammation in the development and progression of these diseases (Stephenson et al., 2018; Hammond et al., 2019). Genetic, histological, and mechanistic studies have all brought forth evidence that dysregulation of normal immune pathways can lead to an increase in neurotoxic pro-inflammatory cytokine production, untethered immune cell reactivity and proliferation, and altered phagocytic capabilities, all of which can contribute to the disease states associated with many of these conditions. Furthermore, although immune dysfunction was once thought of as secondary to the underlying primary disease mechanism, recent evidence suggests that immune dysfunction can play a central role in the development of many neurodegenerative diseases (Lucin and Wyss-Coray, 2009; Heneka et al., 2014; Ising and Heneka, 2018). Understanding the immune processes that are shaped by cytokine, chemokine, and growth factor production is an important aspect of therapeutic development for many disease states. Therefore, it is vital for researchers to investigate the roles of normal and dysfunctional immune signaling pathways during neurodegenerative disease to determine novel targets for altering disease development and progression.

Of particular interest in recent studies of neurodegenerative disease is the role of the chemokine fractalkine (CX_3_CL1, FKN) and its cognate receptor (CX_3_CR1). FKN is a unique transmembrane protein that is constitutively expressed by neurons and endothelial cells (Bazan et al., 1997; Harrison et al., 1998). FKN is unique in that it exclusively signals through its receptor, CX_3_CR1, which is expressed on certain leukocytes including monocytes, macrophages, and microglia (Julia, 2012; White and Greaves, 2012). Functionally, FKN acts as both a chemotactic molecule for the CX_3_CR1-expressing immune cells for extravasation to areas of tissue damage, as well as promote adhesion of circulating immune cells to the endothelium (Haskell et al., 1999; Hermand et al., 2008). In addition to its chemokine functions, FKN signaling has a role in dampening microglial activation in the diseased brain (Paolicelli et al., 2014), and also regulate neuron homeostatic functions and maintenance, synapse plasticity, and synapse pruning during development (Cardona et al., 2006; Paolicelli et al., 2011). Based on FKN’s involvement in controlling the inflammatory process, its role in disease states of tissues expressing high levels of resident immune cells needs to be further explored.

Classically, the inner ear has been thought of as immune privileged, due to the presence of a blood-labyrinth barrier, analogous to the blood-brain barrier in the CNS, and lack of lymphatic drainage (Harris, 1983, 1984; McCabe, 1989). However, over the past couple of decades, as better animal models and imaging techniques have been developed, studies have shown that in response to noise trauma, ototoxic drug administration, normal aging (Frye et al., 2017; Noble et al., 2019), or genetic manipulation, the epithelial, neuronal, and mesenchymal regions of the cochlea experience an augmented immune and inflammatory response. Furthermore, under static conditions, a population of tissue-resident macrophages, analogous to the microglia of the CNS, reside in the cochlea that become activated following insult (Lang et al., 2006; Okano et al., 2008; Sato et al., 2008; Shi, 2010). In addition, following cochlear trauma, these resident macrophages may play a critical role in the recruitment of immune cells from the blood circulation to provide support during the inflammatory process (Hirose et al., 2005). Despite growing evidence, the signaling mechanisms responsible for immune cell activation and recruitment and in particular, the functions of immune cells in the injured cochlea and hearing loss, remains unclear.

In this review, we aim to better understand FKN signaling and how it relates to immune activation during neurodegenerative disease development and progression. Importantly, we will review the evidence of immune activation, specifically through FKN signaling, as being oto- and neuroprotective in the injured cochlea. This will include evaluation of the emerging role of macrophages and FKN signaling regulation in promoting sensory hair cell and neuron viability and spontaneous repair of cochlear ribbon synapses following Sensorineural hearing loss (SNHL). Finally, we will postulate further work that needs to be conducted to determine the mechanisms by which macrophages and FKN signaling mediate neuroprotection during cochlear damage, to harness these protective capabilities clinically.

## Cochlear Macrophages

First discovered by Elie Metchnikoff in the late 19th century, macrophages are the main effector cells of innate immunity that are derived from precursor monocytes (Tauber, 2003; Cooper and Alder, 2006; Epelman et al., 2014). The main function of macrophages, or “big eaters,” is to phagocytose or engulf foreign material or cellular debris during injury to maintain tissue homeostasis and promote wound repair (Mosser et al., 2021). Macrophages also participate in antigen presentation to adaptive immune cells such as T cells, production of reactive species, trophic molecules, cytokines, and chemokines to promote or resolve the inflammatory process, as well as play vital roles in tissue development and physiology (Gordon and Martinez-Pomares, 2017; Gordon and Pluddemann, 2017). Despite the known protective functions of macrophages, under certain conditions macrophages can be toxic through the excess uncontrolled production of reactive oxygen species (ROS) and reactive nitrogen species (RNS) and pro-inflammatory mediators or non-selective phagocytosis of healthy cells due to dysregulation in the resolution of macrophage inflammatory and phagocytic response (Arango Duque and Descoteaux, 2014; Dantzer, 2018; Oved et al., 2019). This dichotomy of macrophages having a role in protection and damage has led to significant research about macrophage activation and function in various disease development and progression.

### Macrophages in a Developing Cochlea

Studies using mouse models have shown that in a developing cochlea, macrophages originate from two sources, the yolk sac and the fetal liver (Ginhoux and Guilliams, 2016; Kishimoto et al., 2019). These two separate lineages are distinguished by the dependence of colony stimulating factor 1 (*Csf1*) which is a cytokine required for differentiation of hematopoietic stem cells into macrophages and labels yolk sac-derived macrophages. Alternatively, fetal liver-derived macrophages differentiate from the cluster of differentiation molecule 11b (*CD11b*) expressing progenitor monocytes. Yolk sac-derived macrophages originate around mouse embryonic day 7.5 (E7.5) and travel to the otocyst around mouse E10.5. Fetal liver-derived macrophages travel to the developing cochlea around mouse E14.5 (Ginhoux and Guilliams, 2016). Studies in *Csf1*- knockout (KO) mice revealed drastically reduced density of macrophages in the spiral ligament and spiral ganglion, suggesting that these macrophages predominantly originate from the yolk sac (Ginhoux et al., 2010; Hoeffel et al., 2012). Cochlear macrophages have been shown to self-renew throughout their lifetime (Hashimoto et al., 2013) and can also be slowly replaced by bone-marrow-derived circulating monocyte-derived macrophages (Liu et al., 2018). However, whether it is the yolk sac- and fetal liver-derived macrophages that establish embryonically persist or are replaced by bone-marrow-derived monocyte-derived macrophages in an adult cochlea, remains to be established. During development, cochlear macrophages expand, peak during neonatal stages, and decline after P3 (Kishimoto et al., 2019). Nevertheless, the mechanisms of macrophage colonization, expansion, and maintenance in both developing and adult cochlea are completely unknown.

During development, studies have found that aside from their known function of immune surveillance and phagocytosis, macrophages may play an important role in cochlear ribbon synapse formation through synaptic pruning, akin to what microglia do in the brain (Dong et al., 2018; Coate et al., 2019). This is supported by data showing the presence of macrophages near ribbon synapses throughout postnatal cochlear development (Dong et al., 2018). In addition, macrophages play a role in spiral ganglion neuron (SGN) development, through the clearance of apoptotic neuronal precursors during development (Echteler et al., 2005). Together, these data suggest that macrophages may play a vital role in cochlear development. Further understanding of the role of macrophages during cochlear development could help to determine therapeutic targets for SGN protection or regeneration and cochlear ribbon synapse regeneration following trauma.

### Macrophages in the Cochlea During Steady-State and Pathology

In a mature cochlea, under steady-state, a resident population of macrophages can be found in multiple regions, including osseous spiral lamina (OSL), stria vascularis (SVA), spiral ligament, spiral limbus, basilar membrane, and spiral ganglion (Hirose et al., 2005). These resident macrophages are thought to play a vital role in maintaining tissue homeostasis, as well as being the main cells responsible for initiating an immune response following cochlear injury (Okano and Kishimoto, 2019). Notably, under steady-state, macrophages are absent from the organ of Corti, in which reside the sensory hair cell receptors and non-sensory supporting cells. According to Hirose et al. (2005) in 2005, this lack of macrophages in the organ of Corti could be attributed to the high potassium (K^+^) content of cochlear endolymph, creating an environment that is likely toxic for macrophage survival. Under normal conditions, resident macrophages in the cochlea display site-specific morphology. Apical macrophages are characterized by dendritic morphology with long and thin processes (pseudopodia). This dendritic morphology is characteristic of macrophages in surveillance mode (Nimmerjahn et al., 2005; Cao et al., 2015; Gordon and Pluddemann, 2017). Macrophages in the middle turn of the cochlea are characterized by a transitional morphology, between dendritic and amoeboid shapes. This amoeboid shape is characteristic of an activated macrophage (Hirayama et al., 2017). Finally, basal region macrophages are found to have an amoeboid morphology (Yang et al., 2015; Frye et al., 2017). Beyond morphology, the main question surrounding cochlear macrophages during steady-state is how they are maintained throughout life. Data from Okano and Kishimoto (2019) suggests that intact *Csf1* signaling plays a major role in cochlear macrophage maintenance during life. However, the precise mechanisms of how cochlear macrophages maintain themselves during steady-state, how macrophages interact with other cochlear cells, and what specific function macrophages have in the cochlea during steady-state is largely unknown and requires further investigation.

Following cochlear sensory hair cell damage caused by ototoxic drug toxicity or noise trauma, there is a significant increase in the numbers of macrophages, likely due to infiltration of macrophages from the blood vasculature (Fredelius and Rask-Andersen, 1990; Hirose et al., 2005; Zhang et al., 2012; Fujioka et al., 2014; Kaur et al., 2015, 2018, 2019; Tan et al., 2016; Wood and Zuo, 2017). To address whether such an increase is due to hair cell loss alone, Kaur et al. (2015) using the *Pou4f3-huDTR* mouse model that expresses the human diphtheria toxin receptor (*huDTR*) on POU class 4 transcription factor 3 *(Pou4f3)* expressing inner hair cells (IHCs) and outer hair cells (OHCs) to selectively ablate hair cells upon diphtheria toxin (DT) administration (Golub et al., 2012; Tong et al., 2015), found that selective hair cell ablation without additional trauma to the sensory epithelium or spiral ganglion was sufficient to increase macrophage numbers in the injured cochlea. Evidently, macrophage numbers increased in the sensory epithelium at the same time when hair cells begin to die (i.e., ~3 days after DT administration). Interestingly, macrophage numbers also increased in the spiral ganglion at ~7 days after DT administration without any evident loss of SGNs. More recently, it has been shown that macrophages’ density increases in the IHC-synaptic region following moderate synaptopathic noise trauma that does not lead to any evident hair cell death (Kaur et al., 2019). Together, these studies suggest that there is a distinct temporal and spatial distribution pattern of macrophages and that macrophage response is both specific and non-specific to hair cell death following cochlear trauma. In addition, studies have shown that these infiltrating macrophages, in coordination with other cells in the cochlea, may instigate the transition to a pro-inflammatory environment, through the production of proinflammatory mediators, such as cytokines and ROS (Lang et al., 2016). However, the molecular signals regulating macrophage activation, migration, and infiltration from the blood circulation and the precise role of these macrophages in the cochlea after damage remains elusive (He et al., 2020). For the remainder of this review, we will focus on research pointing to the neuroimmune FKN signaling potentially playing a vital role in macrophage infiltration and regulation during cochlear trauma, as well as research that suggests a neuroprotective role for FKN signaling in the injured cochlea.

## The Chemokine Fractalkine and Its Signaling

Chemokines are a family of small chemoattractant cytokines, or signaling proteins, secreted by cells to induce migration of leukocytes from blood into the tissue and vice versa and in the induction of cell movement in response to chemical gradients, a process known as chemotaxis. There are more than 50 known chemokines that play a role in inflammation or homeostasis (Hughes and Nibbs, 2018; Mollica Poeta et al., 2019). Under pathological conditions, any stimulus that results in an altered state of cellular homeostasis can result in the release of chemokines promoting the recruitment of immune cells to sites of inflammation. This recruitment occurs *via* receptor signaling through complementary chemokine receptors that belong to the vast family of G-protein coupled receptors present on innate immune cells (Zlotnik et al., 2006). There are two main families of known chemokine receptors: the conventional chemokine receptors (cCKRs), which include 18 known receptors that mainly signal through G-proteins, and atypical chemokine receptors (ACKRs), which include four receptors that do not use signal transduction pathways associated with cCKRs. cCKRs are associated with cell migration and adhesion while ACKRs are associated with regulation of inflammation, specifically through acting as a chemokine scavenger, promoting chemokine transcytosis, and regulating chemokine gradient formation (Salvi et al., 2017). These chemokine receptors are heptahelical surface molecules, containing seven transmembrane domains, and average around 40 kDa in size (Roy et al., 2014). There are four subclasses of chemokines, differentiated by their cysteine structural motifs (C, CC, CXC, CX_3_C), with the X representing the presence of an amino acid (Zlotnik and Yoshie, 2000).

**Figure 1:**
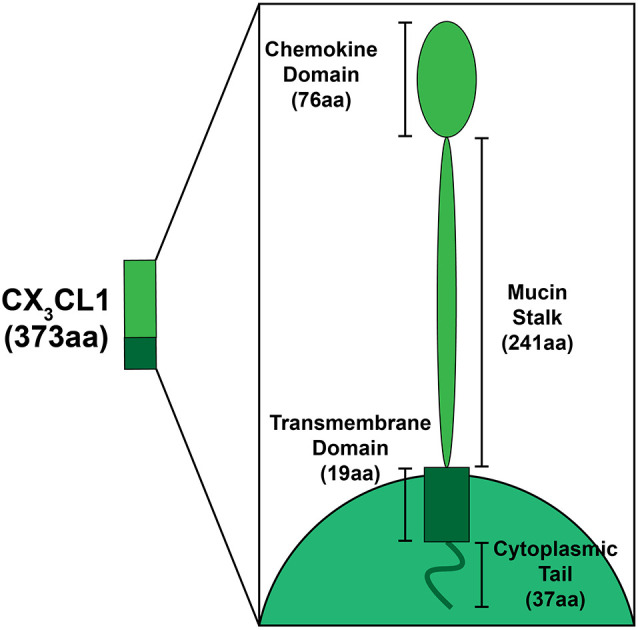
Schematic depicting the structural components of fractalkine (FKN; CX_3_CL1). FKN is a large (373aa) transmembrane protein found exclusively on neurons in the central nervous system (CNS)/peripheral nervous system (PNS), and on SGNs and inner hair cells in the cochlea. Structural components that form FKN include the extracellular chemokine domain (76aa), extracellular mucin stalk (241aa), the transmembrane domain (19aa), and the intracellular cytoplasmic tail (37aa).

**Figure 2:**
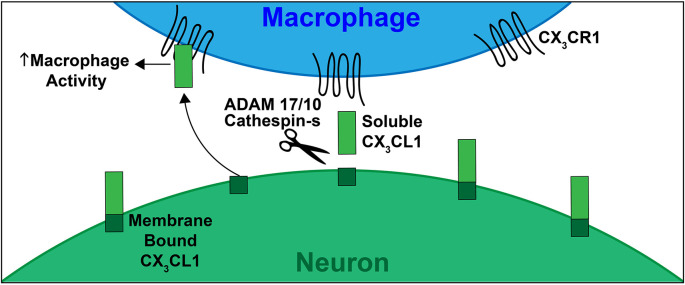
Schematic depicting CX_3_CL1-CX_3_CR1 signaling. CX_3_CL1 of FKN (ligand) is constitutively expressed by neurons in the CNS, PNS and by SGNs and IHCs in the mature cochlea. The CX_3_CR1 (receptor) is present on immune cells, including monocytes, macrophages, and microglia. In response to damage, CX_3_CL1 is proteolytically cleaved from the extracellular side of the neuronal cell membrane via ADAMs 17/10 or Cathespin-s. This cleavage produces the soluble form of CX_3_CL1 (containing the chemokine signaling domain and mucin like stalk). Soluble CX3CL1 binds to its unique receptor CX_3_CR1, resulting in macrophage chemotaxis to sites of injury. The membrane bound form of CX_3_CL1 plays a role in leukocyte adhesion. This signaling, when intact, induces neuroprotection during neuroinflammation and neurodegeneration.

Discovered by Bazan et al. (1997) in the late 1990s, and soon after confirmed by Pan et al. (1997), one of the four subclasses (CX_3_C) is unique in that it only contains one analog, FKN (CX_3_CL1; Koch, 2005). FKN, found constitutively expressed on neurons in the CNS and endothelial cells is unique as it exists both as a soluble protein, which is how most chemokines exist, and also as a cell membrane bound protein (Bazan et al., 1997). In the cochlea, FKN is expressed on SGNs and IHCs (Kaur et al., 2015; Liu et al., 2018). Structurally, FKN is a large transmembrane protein (373 aa) containing an extracellular chemokine domain (76 aa), a mucin-like stalk (241 aa), a transmembrane domain (19 aa), and an intracellular cytoplasmic tail (37 aa; Umehara et al., 2004; Figure 1). The soluble form of FKN is generated by proteolytic cleavage of the mucin-like stalk from the extracellular side of the cell membrane by two members of the disintegrin and metalloprotease family, a disintegrin and metalloprotease domain-17, also referred to as Tumor Necrosis Factor, Alpha (TNF-α), Converting Enzyme (ADAM17/TACE) and ADAM10, or Cathespin-s, a cysteine protease (Hundhausen et al., 2003; Jones et al., 2013). This results in a soluble product containing an N-terminal chemokine domain attached to a mucin-like stalk (Figure 2). ADAM17/TACE cleavage is enhanced by cell stimulation *via* cytokines TNF-α and interleukin-6 (IL-6) signaling, where ADAM10 cleavage is constitutive in unstimulated cells (Hundhausen et al., 2003; O’Sullivan et al., 2016). Cathepsin-s cleavage, like ADAM17/TACE, is associated with pathological conditions, including pain (Clark and Malcangio, 2012).

FKN’s receptor, CX_3_CR1, belongs to the cCKR family and is a class A rhodopsin-like 7-transmembrane G-protein coupled receptor found on leukocytes including monocytes, macrophages, microglia, dendritic cells, NK cells, and a subset of T-cells (Jung et al., 2000; Hughes and Nibbs, 2018; Lee et al., 2018). In the cochlea, CX_3_CR1 is expressed on macrophages (Hirose et al., 2005; Sato et al., 2010). In mice, CX_3_CR1 expression on macrophages is divided into two subsets; chemokine receptor type 2 (*CCR2*) high expressing monocytes have low levels of CX_3_CR1, and *CCR2* low expressing monocytes express high levels of CX_3_CR1 (Geissmann et al., 2003). In humans, this differential expression of CX_3_CR1 is analogous to classical human monocytes (cluster of differentiation (CD), CD14^high^CD16^−^) which express low levels of CX_3_CR1, and non-classical monocytes (CD14^low^CD16^+^) which express high levels of CX_3_CR1 (Geissmann et al., 2003; Meghraoui-Kheddar et al., 2020). CX_3_CR1 exclusively binds to its ligand, FKN (Imai et al., 1997). This is unique to the vast family of chemokines, as of the 50 known chemokines in the body, all except FKN have an affinity for multiple chemokine receptors (Kakinuma and Hwang, 2006). Expression of FKN and CX_3_CR1 is enhanced during inflammatory conditions, suggesting a role for this signaling pathway during inflammatory diseases (Jones et al., 2010).

In the periphery, the membrane-bound form of FKN plays an integral role in leukocyte adhesion to the vascular endothelium. Expression of the membrane-bound form of FKN by endothelial cells provides a high affinity adhesion target, allowing for tight binding to leukocytes despite physiological blood flow (Kerfoot et al., 2003; Schulz et al., 2007). This tight adhesion propagates leukocyte extravasation through the vascular wall and into sites of tissue damage due to the presence of a chemokine gradient. Following proteolytic cleavage, the soluble form of FKN acts as a chemoattractant for leukocytes, including monocytes and macrophages, promoting the recruitment of immune cells to sites of inflammation (Klosowska et al., 2009; Wojdasiewicz et al., 2014). Thus, FKN is considered a “find-me signal” to recruit immune cells to sites of damage (Ravichandran, 2010, 2011).

## The Role of FKN Signaling in Developing and Diseased Brain

In the CNS, FKN plays an important role in neuronal function during physiological conditions (Ransohoff, 2009). In the CNS, FKN is exclusively expressed on neurons and signals through its receptor, CX_3_CR1, expressed exclusively on microglia, the resident macrophages of the CNS (Jung et al., 2000; Cardona et al., 2006). The FKN signaling axis provides microglia with a direct line of communication with the neuronal environment, ensuring homeostatic conditions are preserved during steady-state, and an immune response is mounted during damage or disease. In the adult CNS, microglia play a main role of immune surveillance, mediating the immune response and phagocytosing cellular debris and dead neurons (Colonna and Butovsky, 2017). However, recent studies have shown that microglia also play a major non-immune role in the CNS both during development and during adulthood. These functions range from neurogenesis to synaptic pruning and ultimately play a vital role in the shaping of neuronal circuitry and synaptic plasticity (Schafer and Stevens, 2015). In this section, we will look at the role FKN plays in mediating CNS development, preserving homeostasis during steady-state, and mounting a defense during disease pathology.

### Role of FKN in the Developing Brain

During early development, neurons are forming billions of synaptic connections with each other. However, the number of synaptic connections that are formed far exceed the amount needed for proper brain function. The synapses that are not used must be removed through a process called synaptic pruning, in order to ensure the efficient functioning of the neuronal circuit (Hua and Smith, 2004; Sheridan and Murphy, 2013). Early research by Paolicelli et al. (2011) demonstrated that microglia expressing CX_3_CR1 played a vital role in synaptic pruning during the initial weeks of mouse CNS development, such that disruption of FKN signaling *via* genetic loss of CX_3_CR1 resulted in fewer microglia and excessive synaptic densities on dendritic spines in the hippocampus during postnatal development. Other studies have shown evidence for the role FKN signaling plays in a multitude of vital components to CNS development, including neuronal development and maturation (both through the death of neurons during development, and the protection of neurons during life; Ueno et al., 2013), development of neuronal circuits, and synaptic maturation (Hoshiko et al., 2012; Zhan et al., 2014). The FKN signaling mechanism allows for neuron-glia crosstalk, providing a platform for bidirectional interaction that facilitates communication between neurons and the immune cells that protect them. Research has also shown that after development, microglia can be found in the hippocampus serving in a non-inflammatory role, potentially aiding in synaptic plasticity, and therefore could be important in the processes of learning and memory (Maggi et al., 2011). In addition, constitutive expression of FKN on neurons has been shown to modulate microglial activation, preventing excessive and uncontrolled stimulation which can result in neurotoxicity (Cardona et al., 2006; Biber et al., 2007). Together, these results suggest a vital role for FKN signaling in synaptic remodeling during development.

### Role of FKN in Diseased Brain and Neurodegenerative Diseases

Recent work has suggested that intact FKN signaling plays a critical role in regulating neuronal survival, repair, and regeneration following neuronal injury (Kaur et al., 2015, 2018). Due to FKN being a chemokine and playing an important role in facilitating crosstalk between neurons and microglia, the FKN signaling axis has emerged as an interesting target for researchers studying immune modulation in neurodegenerative diseases. Following the generation of a reporter mouse that replaced endogenous CX_3_CR1 on all cells with green fluorescent protein (GFP), also known as CX_3_CR1^GFP/GFP^ or CX_3_CR1 KO (referred to as CX_3_CR1 global KO for the rest of the manuscript; Jung et al., 2000), researchers have been able to investigate the effects of FKN signal disruption on neuronal function, including during development, aging, and disease. The following sections will explore different neurodegenerative diseases, and how FKN signaling has been implicated in their disease pathology.

#### Glutamate-Induced Excitotoxicity and Epilepsy

Glutamate is the main excitatory neurotransmitter in the brain, playing a pivotal role in the proper physiological function of the CNS (Palmada and Centelles, 1998). Glutamate signals through three types of ionotropic glutamate receptors (GluR), including N-methyl-D-aspartate (NMDA), α-amino-3-hydroxy-5-methylisoxazole-4-propionate (AMPA), and kainic acid (KA; Lauro, 2015). Glutamate signaling *via* these GluRs results in neuronal depolarization and subsequent action potential firing. This process is vital in proper neuronal function, allowing for neuron-to-neuron communication to occur. However, excess glutamate release, leading to over-activation of GluRs, a phenomenon termed glutamate excitotoxicity, can be detrimental and is associated with a number of neurodegenerative diseases, including AD, PD, and various neurological disorders, such as epilepsy (Sheldon and Robinson, 2007; Noda et al., 2011; Lauro, 2015). Glutamate excitotoxicity results in neuronal dysregulation, dysfunction, and death due to increased intracellular calcium levels leading to impaired mitochondrial function and accumulation of oxidizing free radicals. In response to an excitotoxic environment, neurons upregulate, cleave, and release soluble FKN (Chapman et al., 2000). Furthermore, under neuronal injury conditions, intact FKN signaling has been shown to prevent neuronal damage caused by glutamate excitotoxicity (Limatola et al., 2005; Cardona et al., 2006). In these studies, they found that intact FKN signaling resulted in reduced NMDA- or glutamate-mediated elevation of intracellular calcium levels (Deiva et al., 2004; Sheridan et al., 2014) and increased glutamate removal from synaptic clefts *via* glutamate transporter-1 (*GLT-1*) on astrocytes (Catalano et al., 2013). Furthermore, work from Noda et al. (2011) suggests that FKN inhibits excitotoxicity by promoting microglia phagocytosis of damaged neurons and through the production of the antioxidant enzyme heme oxygenase-1 (HO-1). Together, these data suggest that intact FKN signaling protects neurons against glutamate excitotoxicity.

Epilepsy is a neurological disorder highlighted by uncontrolled seizures (Fisher et al., 2005). These seizures are thought to occur due to abnormal hypersynchrony of neuronal action potential firing, due to an imbalance of excitatory and inhibitory neurotransmitters (Dalby and Mody, 2001; Sharma et al., 2007). Furthermore, it has been shown that neurons under epileptic conditions lack γ-aminobutyric acid (GABA) inhibition efficacy, thus leading to an excitotoxic environment (Roseti et al., 2013). Recent work has suggested a role for neuroinflammation in the pathological progression of epilepsy, as studies have shown neuronal hyperactivity trigger microglia process extension and interaction with neurons (Kato et al., 2016; Eyo et al., 2017). Because of the neuroinflammatory role, research has started to investigate the role FKN signaling has in mediating epilepsy, through neuron-microglia interactions. In a 2016 study, Eyo et al. (2016) showed that deficient FKN signaling resulted in increased seizure phenotype. This was attributed to a reduction in microglia-neuron convergence following seizure. In addition, they showed that treatment with FKN resulted in increased microglia-neuron convergence and reduced seizure phenotype, suggesting FKN signaling can mediate neuro-immune interaction following seizures, thus reducing seizure severity. Furthermore, work by Roseti et al. (2013) showed that FKN signaling led to microglia modulation of GABA currents by reducing GABA rundown and promoting GABA current recovery. This modulation has been theorized to occur *via* FKN signaling, promoting a phosphorylation cascade resulting in stabilized GABA receptors on neurons. In this work, they found that FKN signaling modulation of GABA currents counteract neuronal hyperexcitability by preserving inhibitory GABA signaling. Together, these data suggest that FKN signaling is protective during epilepsy, specifically by promoting neuro-immune communication and modulating GABA inhibitory currents to reduce hypersynchronous firing.

#### Alzheimer’s Disease

AD is a progressive neurodegenerative disease affecting memory and cognitive functions. AD is hallmarked by molecular and cellular changes in the brain resulting in neuronal death. These include abnormal levels of extracellular amyloid beta plaque deposition resulting in disruption of neuronal crosstalk and intracellular hyperphosphorylation of the major microtubule associated protein tau forming toxic neurofibrillary tangles within neurons (Finneran et al., 2019). It has also been found that AD patients suffer from chronic neuroinflammation due to dysfunctional microglia. FKN expression in the hippocampus and cortex is drastically reduced in an AD brain (Cho et al., 2011). Paradoxically, conflicting studies have shown both beneficial and detrimental effects of knocking out CX_3_CR1 in terms of AD development and progression. In one study, lack of CX_3_CR1 resulted in diminished amyloid beta plaque formation and increased microglia phagocytosis (Liu et al., 2010). Alternatively, in a mouse model overexpressing human tau (hTau), disruption of FKN signaling exacerbated pathology leading to increased neurofibrillary tangle aggregation (Lee et al., 2014). Interestingly, recent studies have shown that tau can potentially bind to CX_3_CR1, acting as an antagonist against FKN signaling by preventing CX_3_CL1 from binding to its cognate receptor (Bolos et al., 2017). Studies have shown that FKN signaling has anti-inflammatory properties during neurodegenerative diseases, suggesting attenuated FKN signaling could result in diminished anti-inflammatory cytokine production by microglia. Therefore, in this study, FKN signaling antagonism seems to prevent neuronal-microglia crosstalk and subsequent microglia overactivation, resulting in a reduction of anti-inflammatory signaling, and progression of neuroinflammation seen during AD (Finneran and Nash, 2019). This current evidence suggests a duality in the effects of FKN signaling during AD, with both neuroprotective and neurotoxic outcomes at different points during pathology development.

#### Parkinson’s Disease

PD is a neurodegenerative disease characterized by loss of dopaminergic neurons (DA) in the substantia nigra pars compacts (SNpc) of the midbrain, resulting in severe motor, balance, and coordination deficits. In PD, loss of dopaminergic neurons is precipitated by intraneuronal aggregation of α-synuclein, commonly referred to as Lewy bodies (Polymeropoulos et al., 1997; Ross et al., 2008), resulting in neuronal damage and death. Neuroinflammation has been discovered to play an integral function in PD pathogenesis, as microglia dysfunction has been found to exacerbate dopaminergic neuron degeneration. In multiple models of PD, increasing the concentration of soluble FKN has proven to be neuroprotective by inhibiting microglial activation, downregulating pro-inflammatory cytokine production, and protecting DA neurons from degeneration (Cardona et al., 2006; Pabon et al., 2011; Morganti et al., 2012). Additionally, in a study using the CX_3_CR1 global KO mouse model, where CX_3_CR1 is replaced by GFP on all cells expressing CX_3_CR1, Cardona et al. (2006) showed that disruption in FKN signaling resulted in an increased loss of neurons in the 1-methyl-4-phenyl-1,2,3,6-tetrahydropyridine (MPTP) model of PD. Finally, studies have shown that in PD models overexpressing α-synuclein, FKN diminished DA neurodegeneration (Nash et al., 2015; Thome et al., 2015). Taken together, these results suggest FKN signaling is neuroprotective in PD.

#### Multiple Sclerosis

MS is a neurodegenerative demyelinating disease resulting from the destruction of myelin and oligodendrocytes insulating neurons in the brain and spinal cord. Demyelination of neuronal axons results in diminished neuronal signal firing, leading to symptoms ranging from motor, sensory, and cognitive dysfunction. Endpoint pathology of MS includes neuronal degeneration resulting in paralysis (Ridderstad Wollberg et al., 2014). While the direct cause of MS is still unknown, recent studies have shown evidence that immune dysfunction plays a key role in disease development (Cardona et al., 2018). However, in MS, microglia have been found to play an integral role in myelin destruction. This is supported by work showing that in a model of autoimmune encephalomyelitis (EAE), CX_3_CR1 deficient mice have enhanced demyelination and subsequent neuronal damage (Garcia et al., 2013). Interestingly, there is little to weak evidence to suggest that FKN signaling acts as a “find me” signal for microglia in the brain. Rather, data suggests that FKN signaling functions as a checkpoint, preventing the uncontrolled activation of microglia (Cardona et al., 2006). In these studies, when FKN signaling is disrupted, this checkpoint is removed, and microglia pro-inflammatory and phagocytic activity becomes dysregulated, leading to neurotoxicity. Therefore, this evidence supports data suggesting a neuroprotective role for microglia induced by intact FKN signaling during MS-associated demyelination.

#### Retinal Neurodegeneration

Diabetic retinopathy (DR) is a common microvascular disorder associated with diabetes and a leading cause of blindness worldwide (Antonetti et al., 2012). In this disease there is a breakdown of the blood-retinal barrier, leading to loss of cells in the retina (Barber, 2003). Although the etiology of the disease is not well understood, uncontrolled retinal cell death will eventually lead to vision loss and blindness. Interestingly, a number of studies have observed microglia activation in the early stages of DR, promoting the production and release of proinflammatory cytokines, exacerbating the development of retinal cell death (Liang et al., 2009; Tang and Kern, 2011). Because of this, the FKN signaling axis has been examined for its ability to control neuroimmune signaling between retinal neurons and microglia, and thus prevent over-activation of microglia. In 2015, Cardona et al. (2015) crossed the Insulin2^Akita^ mouse model of DR with CX_3_CR1 deficient mice to determine the role FKN signaling plays in DR pathology. In this study, they found that the DR mouse without intact FKN signaling had microglia with prolonged activity in the retina. This prolonged activity was accompanied by increased retinal ganglion cell (RGC) death and increased levels of proinflammatory interleukin 1 beta (IL-1β). These results suggest that in a DR mouse model, intact FKN signaling is neuroprotective towards RGCs by reducing microglia activity and diminishing proinflammatory mediator production. Furthermore, in a 2016 study by Mendiola et al. (2016), they found that in the same mouse model of DR, intact FKN signaling reduced the clustering of microglia around retinal microvasculature, and in turn prevented vascular leakage and edema, a hallmark of DR development. In addition, when FKN was disrupted, they again found that there was an increased production of proinflammatory IL-1β by microglia and astrocytes in the retina. This supports the previously mentioned results in a model of MS, where intact FKN signaling acts as a checkpoint to prevent neurotoxic overactivation of microglia, including the chronic production of pro-inflammatory mediators.

Retinitis pigmentosa (RP) is a genetic mutation disease characterized by the progressive degeneration of photoreceptors and is a main cause of blindness worldwide, specifically in children (Hartong et al., 2006). Evidence suggests that the development and progression of RP are associated with microglia activation. Peng et al. (2014) in 2014 found that in the rd10 mouse model of RP, disrupted FKN signaling resulted in increased photoreceptor degeneration, caused by increased microglia activation. In addition, in 2016, using the same RP mouse model, Zabel et al. (2016) found that this increased photoreceptor degeneration was caused by increased microglia phagocytosis and increased production of proinflammatory cytokines by microglia. This microglia activity was found to be controlled by the FKN axis, where when intact, reduced photoreceptor degeneration was observed.

Glaucoma is a progressive neurodegenerative disease of the eye resulting in irreversible vision loss, precipitated by RGC death (Almasieh et al., 2012; Nickells et al., 2012). Although little is known about the molecular mechanisms responsible for glaucoma development, growing evidence suggests a neuroinflammatory role mediated by microglia (Cui et al., 2009; Ebneter et al., 2010; Bosco et al., 2011). Therefore, in 2014 Wang et al. (2014) performed a study to determine the role FKN signaling had in modulating retinal microglia activation during glaucoma. In this study they found that disruption of FKN signaling enhanced the neurotoxic behavior of retinal microglia, leading to increased RGC death. Furthermore, they found that intact FKN signaling prevented microglia activation in the retina, thus increasing RGC survival. Together, these studies suggest that intact FKN signaling plays a neuroprotective role in the diseased retina by decreasing microglia neurotoxicity and enhancing RGC and photoreceptor survival.

#### Human CX_3_CR1 Polymorphisms and Neurodegeneration

Polymorphisms in CX_3_CR1 have been attributed to a number of inflammatory diseases, including age-related macular degeneration (AMD) and MS. CX_3_CR1 has two single nucleotide polymorphisms (SNPs) in humans: V249I and T280M (Faure et al., 2000; Chan et al., 2005). These polymorphisms are present in approximately 20% of the population, and result in reduced affinity for FKN to its receptor, leading to ineffective signaling (McDermott et al., 2003; Cardona et al., 2018) or reduced expression of CX_3_CR1 (Chan et al., 2005). Due to the resultant defective neuronal-glia communication, these SNPs have been studied for their effects in the development and progression of many inflammatory diseases.

AMD is one of the most common age-related eye diseases leading to vision loss. In this disease, there is a progressive degeneration of the macula in the retina, leading to loss of the center field of vision. This is either termed dry AMD, where the retina itself deteriorates, or wet AMD where leaky blood vessels grow under the retina. Although the etiology is largely unknown, the development of AMD is heavily linked with a combination of environmental and genetic factors. One such genetic factor that confers increased susceptibility to AMD is CX_3_CR1 SNPs (Tuo et al., 2004; Chan et al., 2005; Ma et al., 2015). Possible explanations for CX_3_CR1 SNP susceptibility for developing AMD could be attributed to the significant role macrophage activity has been shown to play in the pathogenesis of AMD (Ambati et al., 2003). Specifically, through over-activity and improper immune activation, resulting in excessive inflammation and uncontrolled phagocytosis of retinal cells. Thus, altered neuro-immune communication, through reduced FKN affinity or reduced CX_3_CR1 expression caused by CX_3_CR1 SNPs, could lead to altered immune cell activation and increased susceptibility to developing AMD.

In addition to AMD, studies have investigated the role of CX_3_CR1 SNPs in the susceptibility of developing MS. Studies have shown that there are significantly different levels of CX_3_CR1 expression in MS patients compared to healthy individuals (Infante-Duarte et al., 2005). Thus, this suggests a role for CX_3_CR1 in the development of MS. In a 2011 study by the International Multiple Sclerosis Genetics Consortium and the Wellcome Trust Case Control Consortium et al. (2011) using a genome-wide association study (GWAS) of 9,772 cases, they sought to determine any genetic risk factors associated with MS development. Although they found numerous genetic risk factors associated with MS, CX_3_CR1 SNPs were not implicated. Furthermore, in studies of MS patients expressing single nucleotide polymorphisms in the CX_3_CR1 locus (CX_3_CR1^M280^), Stojkovic et al. (2012) found no association of CX_3_CR1 SNPs with MS susceptibility. Interestingly, in this study, CX_3_CR1 SNPs seemed to confer protection in MS patients, preventing the switch to a more progressive form of the disease. This could potentially be due to reduced demyelination caused by the modulation of microglia activity. However, in a mouse model of EAE, used to emulate MS disease progression in mice, disrupted FKN signaling through the CX_3_CR1^M280^ SNP, exacerbated EAE progression, including microglia dysfunction, and subsequent inflammation, demyelination, and degeneration of neurons (Cardona et al., 2018). Thus, in a mouse model of EAE, CX_3_CR1 SNPs seem to exacerbate demyelinating pathology, suggesting FKN genetic variants indeed confer MS susceptibility. These conflicting results can most likely be explained by the differing models used to explore MS susceptibility, however, more work needs to be done to determine the role CX_3_CR1 SNPs signaling has in MS susceptibility, development and progression.

## Emerging Roles of Fractalkine Signaling in The Injured Cochlea

SNHL occurs due to loss of the sensory hair cells, IHC-SGN ribbon synapses, or loss of SGNs. Such loss can occur due to sterile traumas such as loud and prolonged noise exposure and ototoxic drug administration, infection, or normal physiological processes such as aging. Until recently, it was not known whether there was an inflammatory component in SNHL development and progression. In tissues throughout the body, macrophages play a key role in cellular development, homeostasis, and disease development and progression. However, it is not clear whether macrophages play a similar role in the cochlea during physiological and pathological conditions. To determine the role of macrophages in cochlear pathology and SNHL, one must understand the mechanisms vital for immune cell communication within the cochlea. In the following sections, we will look at emerging evidence showing that FKN signaling between IHCs and SGNs that express FKN ligand and cochlear macrophages that express CX_3_CR1 receptor is chemotactic and neuroprotective following cochlear injury.

### FKN as a Macrophage “find-me” Signal in the Injured Cochlea

FKN is a chemokine that is constitutively expressed by IHCs and SGNs in the mature cochlea and its level increases following cochlear trauma (Sato et al., 2010; Kaur et al., 2015; Sun et al., 2015). Is FKN the chemotactic molecule that allows local or blood circulating macrophages to locally migrate or infiltrate from the circulation into the damaged cochlea? To address this, numerous studies have used the mouse that lacks CX_3_CR1 (CX_3_CR1 global KO) on all cells as a model to disrupt FKN signaling to understand its function in macrophage chemotaxis in the injured cochlea (Jung et al., 2000). In 2010, Sato et al. (2010) treated CX_3_CR1 global KO (disrupted FKN signaling) and CX_3_CR1 heterozygous (intact FKN signaling) mice with aminoglycoside kanamycin to induce ototoxicity. The number of macrophages increased after kanamycin-induced hair cell death however, the degree of increase was not significantly different between CX_3_CR1 heterozygous and KO mice, and rather was found to be higher in the spiral ligament in certain cochlear frequency locations of the CX_3_CR1 global KO mice (Tuo et al., 2004). Another study in 2008 by Sato et al. (2008) reported similar findings following loud acoustic trauma. Similarly, Kaur et al. (2019) in 2019 observed in a model of mild acute acoustic trauma (90 dB SPL, 8–16 kHz, 2 h), that absence of CX_3_CR1 on macrophages did not alter the density of resident macrophages that migrated into the noise-damaged IHC-synaptic region when compared to mice with intact FKN signaling. On the contrary, in a 2015 study, Kaur et al. (2015) reported that disruption of FKN signaling in CX_3_CR1 global KO mice resulted in a significant reduction in the macrophage density in both sensory epithelium and spiral ganglion after selective hair cell ablation. Furthermore, in 2018 Kaur et al. (2018) observed a 36% reduction in macrophage density in the sensory epithelium following kanamycin-furosemide treatment when FKN signaling was disrupted. Interestingly, in this study, they observed that disruption of FKN signaling had no significant effect on macrophage density in the spiral ganglion following kanamycin treatment, or anywhere in the cochlea following very loud acoustic trauma. However, at this time, the origin of these macrophages following cochlear trauma is unclear. In their 2015 study, Kaur et al. (2015) addressed macrophage origin following cochlear trauma by using BrdU labeling to determine if resident macrophages proliferate after selective hair cell ablation. In this study, no BrdU labeled macrophages were reported suggesting that resident macrophages do not proliferate and that the increase in macrophage density following trauma is a result of infiltration of macrophages from the circulation. Therefore, the conflicting results of the aforementioned studies suggest that perhaps FKN signaling act as a “find-me” signal for the blood circulating macrophages, but not for resident (local) macrophages in the injured cochlea and that such FKN-mediated macrophage chemotaxis could be dependent on the type and extent of the cochlear injury. Additionally, these results could also suggest an important role for additional neuroimmune signaling pathways, including CD200/CD300R and CD47/SIRP, in macrophage activity following cochlear trauma (Marinelli et al., 2019). Further work using fate-mapping or lineage tracing tools that distinguishes resident and recruited macrophages is required to determine whether FKN signaling regulates the chemotaxis of resident or recruited macrophages in the injured cochlea. Furthermore, work characterizing the activity of additional neuroimmune signaling pathways needs to be done to determine the extent of FKN signaling role in macrophage activity within the damaged cochlea. It is important to note that disruption of FKN signaling due to the absence of CX_3_CR1 on macrophages did not affect macrophage numbers in the normal cochlea (Sato et al., 2008, 2010; Kaur et al., 2018, 2019), suggesting that this signaling is not critical for the maintenance and survival of cochlear resident macrophages.

### FKN Promotes the Survival of Sensory Hair Cells in the Injured Cochlea

The most common histological evidence of SNHL is loss of cochlear sensory hair cells or damage to their stereocilia bundles. Loss of hair cells or hair cell function prevents the transduction of environmental sound waves into receptor (IHC) potentials which is necessary for the release of excitatory glutamate from IHC. This, in turn, inhibits action potential generation in afferent cochlear SGN fibers, and therefore hearing is lost (Liberman, 1984). With recent evidence suggesting a neuroinflammatory element in SNHL, understanding the mechanisms responsible for communication between cochlear hair cells and immune cells could be vital in determining therapeutic targets for hair cell protection following cochlear trauma. In a model of aminoglycoside ototoxicity, Sato et al. (2010) showed that intact FKN signaling protected cochlear hair cells. Here, following kanamycin treatment, disruption of FKN signaling, due to genetic deletion of CX_3_CR1 (using the CX_3_CR1 global KO mouse model), resulted in a significant loss of OHCs in the 5–16 kHz range of the cochlea. This loss of OHCs correlated to elevated ABR thresholds in CX_3_CR1 global KO animals in the same tonotopic cochlear regions (Sato et al., 2010). Sato et al. (2008) also found similar results after acoustic trauma. Similarly, in 2019, Kaur et al. (2019) showed that following a mild synaptopathic noise trauma (90 dB SPL, 8–16 kHz, 2 h), lack of CX_3_CR1 resulted in damage and loss of IHCs, specifically from the middle and basal region of the cochlea (Kaur et al., 2019). Together, these results suggest that intact FKN signaling is protective towards OHCs and IHCs following cochlear trauma, including acoustic damage and aminoglycoside drug toxicity. Further research work is needed to determine the specific mechanisms responsible for FKN-signaling mediated inner and outer hair cell protection following cochlear trauma, especially when FKN is only expressed by IHCs and not OHCs (Sato et al., 2010; Kaur et al., 2015).

### FKN Promotes Survival of Spiral Ganglion Neurons in the Injured Cochlea

Historically, many studies have demonstrated that degeneration and loss of SGNs follow the death of sensory hair cells due to acoustic trauma or ototoxic drugs (secondary degeneration; Dupont et al., 1993; Gillespie and Shepherd, 2005). However, recent studies have shown that SGNs can die without hair cell loss (primary degeneration; Stankovic et al., 2004; Kujawa and Liberman, 2009). Both primary and secondary SGN degeneration are gradual processes, with peripheral axon degeneration occurring within days and SGN somata death occurring within weeks, with some studies even suggesting SGNs can survive months to years post-trauma (Zilberstein et al., 2012). Beyond our current understanding of the protective roles played by neurotrophic factors released by supporting and glial cells and intrinsic electrical activity of SGNs, our understanding of the non-neuronal factors that regulate SGN degeneration and survival in the damaged cochlea is limited. In their 2015 work, the Kaur group (Kaur et al., 2015) showed that in the *Pou4f3huDTR* mouse model of selective hair cell ablation, disruption of FKN signaling, due to genetic lack of CX_3_CR1 receptor on macrophages, resulted in an increase in SGN death at 2 months after selective hair cell ablation compared to animals with intact FKN signaling. Interestingly, this increase in SGN death was observed throughout the cochlea, and directly correlated with fewer numbers of macrophages present in the spiral ganglia. Furthermore, in their 2018 study, Kaur et al. (2018) examined whether genetic disruption of FKN signaling would affect SGN survival in clinically and biologically relevant mouse models of cochlear trauma. Here, they utilized a model of loud acoustic trauma, where mice were exposed to 8–16 kHz octave band noise at 120 dB SPL for 2 h (Wang et al., 2002). In this model, noise trauma results in permanent hearing loss, resulting from widespread hair cell loss 7-days post-noise exposure. Following acoustic trauma, they observed approximately a 29% reduction in SGN density in animals with disrupted FKN signaling (CX_3_CR1 global KO) when compared to animals with intact FKN signaling. Additionally, in this study, they also used a model of Kanamycin/Furosemide-induced ototoxicity (KF; Oesterle et al., 2008; Hirose and Sato, 2011; Hirose et al., 2014). In this model, administration of KF caused significant hearing loss resulting from widespread hair cell death 14 days following drug administration. Following KF treatment, disrupted FKN signaling caused by genetic deletion of CX_3_CR1 resulted in a ~31% reduction in SGN density when compared to CX_3_CR1 heterozygous controls that have intact FKN signaling. Importantly, in both biological models of SNHL, reduction in SGN survival correlated to the reduced density of macrophages in the spiral ganglia of the injured CX_3_CR1 global KO mice. Finally, in their 2019 study, Kaur et al. (2019) demonstrated that following mild acute acoustic trauma that did not cause any evident hair cell loss or any significant increase in macrophage numbers in the spiral ganglia, the absence of CX_3_CR1 on macrophages still resulted in a robust and gradual SGN death. Of note, the lack of CX_3_CR1 alone did not cause loss of SGNs in any of these above-mentioned studies. Interestingly, the precise mechanisms by which intact FKN signaling, and macrophages promote SGN survival in the injured cochlea remains unknown and are under investigation. However, FKN signaling seems to play dual roles, as a chemokine, to recruit anti-inflammatory and pro-healing circulating macrophages, and as checkpoint molecule, to regulate the inflammatory and phagocytic phenotype of resident and circulating macrophages. However, more work needs to be done to determine the precise mechanisms of FKN-mediated SGN neuroprotection.

Kaur et al. (2018) further demonstrated an increase in the expression of pro-inflammatory cytokine IL-1β in the spiral ganglion and macrophages of mice lacking CX_3_CR1 compared to mice with intact CX_3_CR1 following aminoglycoside ototoxicity (Kaur et al., 2018). Similarly, studies in the CNS have found that altered FKN signaling affects microglia production of cytokines, including the enhanced expression of IL-1β (Cardona et al., 2006, 2015). Together, these studies suggest that macrophages serve a neuroprotective role in the injured cochlea *via* FKN signaling possibly by regulating the production of pro-inflammatory mediators from cells in the cochlea including macrophages. This is in line with previously mentioned studies of neurodegenerative diseases, where FKN signaling affects macrophage activity, acting as a checkpoint to prevent microglia overactivation (Cardona et al., 2006). Furthermore, this is in line with previously mentioned contradictory evidence where studies have shown both beneficial and detrimental effects of FKN signaling and macrophage/microglia activity or phenotype during neurodegenerative disease development and pathology, ultimately suggesting a context-dependent role in the cochlea. Regardless, whether these pro-inflammatory molecules are toxic to SGNs survival after cochlear injury needs validation.

### FKN Promotes the Repair of Damaged Ribbon Synapses

The idea of “hidden hearing loss” was first described by Kujawa and Liberman (2009) in 2009, where they found that a moderate acoustic trauma (100 dB SPL, 8–16 kHz, 2 h) induced a reversible loss of hearing function (*via* auditory brainstem response (ABR) and distortion product otoacoustic emissions (DPOAE) thresholds) without causing sensory hair cell death. Interestingly, despite the complete recovery of ABR and DPOAE thresholds, they found that suprathreshold ABR Wave 1 amplitudes never fully recovered. This was supported by histological analysis showing permanent loss of IHC ribbon synapses, and progressive SGN death. Clinically, the loss of IHC ribbon synapses causing SGN death has profound effects on sound localization and understanding speech in noisy environments (Liberman and Kujawa, 2017). Kaur et al. (2018) reported an immediate increase in macrophage numbers in the damaged IHC synaptic region following synaptopathic acoustic trauma. In their 2019 work, they further examined the effects disruption of FKN signaling has on degeneration and repair of the synaptic connections between IHCs and SGNs (Kaur et al., 2019). Here, they exposed mice with intact FKN signaling and those that lacked CX_3_CR1 to a moderate noise level (90 dB SPL, 8–16 kHz octave band, 2 h). Such noise trauma resulted in a rapid degeneration of ~50% of IHC-ribbon synapses in the mid-basal and basal regions of the cochlea and an increase in macrophage numbers in the IHC synaptic region despite any evident hair cell death. The noise-damaged synapses gradually repaired in mice with intact FKN signaling, however the absence of CX_3_CR1 impaired such spontaneous synaptic repair that correlated with reduced ABR Wave 1 input-output function (amplitudes). Altogether, this work suggests that FKN signaling also plays a vital role in the spontaneous repair of damaged ribbon synapses. However, further work needs to be done to determine the exact roles of macrophages and FKN signaling in ribbon synapse degeneration and repair in order to develop novel immuno-therapies for hidden-hearing loss. In the final section, we will look at some of the work being done examining the therapeutic benefits of FKN overexpression on a variety of neurodegenerative diseases.

## FKN as A Therapeutic Target for Neuroprotection

The previous sections outlined the multitude of studies surrounding the role FKN signaling has in modulating the immune response during neurodegenerative disease development and progression. Due to the role of FKN signaling in microglia/macrophage activation, as well as FKN’s exclusivity for its receptor CX_3_CR1, this signaling axis has the potential to be an intriguing therapeutic target for any disease involving chronic neuroinflammation. In this section, we will investigate some of the work being done on targeting the FKN signaling to treat neurodegenerative diseases.

In 2013, Nash et al. (2013) investigated the role of FKN overexpression by using recombinant adeno-associated virus (AAV) vectors to deliver soluble FKN to reduce tau pathology in a mouse model of tauopathy. AAV serotype 9-expressing soluble FKN (AAV9) increased FKN levels up to two-fold over endogenous levels in the mouse hippocampus and reduced neurofibrillary tangles (Nash et al., 2013). Despite the increase in FKN expression, they did not observe any significant rescue in cognitive function as measured by radial arm water maze, possibly attributed to the aggressive tauopathy associated with the mouse model, or limited transduction of AAV9 vectors in the hippocampus. However, in a 2019 study by Finneran et al. (2019), they found that CNS-wide overexpression of soluble FKN *via* AAV serotype 2 (AAV2) delivery led to a rescue in cognitive function, as seen through novel recognition tasks and radial arm water maze behavior, in the same mouse model of tauopathy. This suggests exogenous FKN expression can rescue pathology associated with tauopathy. Furthermore, a 2011 study by Pabon et al. (2011) found that in the 6-hydroxydopamine (6-OHDA) rat model of PD, striatal delivery of exogenous recombinant FKN proved to be neuroprotective by reducing the size of the dopaminergic lesion, as well as reduced neuronal loss. This was further supported by a 2012 study by Morganti et al. (2012), where AAV9 delivery of soluble FKN was neuroprotective in the MPTP model of PD, treatment with AAV overexpressing FKN resulting in reduced neurotoxic effects. This was through preserved motor functions, reduced neuronal loss, and reduced proinflammatory cytokine release by activated microglia. Also, work led by the Nash group in 2014 found that the use of AAV2 to deliver soluble FKN reduced α-synuclein-mediated neurodegeneration in the MTPT model of PD in rats (Nash et al., 2015). Finally, using a mouse model of RP, Wang et al. (2019) showed that AAV serotype 8 (AAV8) delivery of soluble FKN prolongs cone cell survival and improves visual function. This neuroprotection occurred despite FKN overexpression not reducing cytokine levels or microglia activation in the retina. Although the mechanisms by which FKN overexpression led to neuroprotection in a model of RP remains unknown, these results further support the viability of FKN as a therapeutic target and could have implications for other neurodegenerative diseases of the eye, including AMD, DR, and glaucoma. Together, these examples are just a few studies that have explored the possibility of using FKN overexpression as a therapeutic measure for various neurodegenerative diseases. Although more work needs to be done to determine the therapeutic viability of FKN overexpression, these studies suggest that the use of targeted viral vectors, therapeutic peptides, or potentially pharmacological CX_3_CR1 receptor agonists, to target the FKN signaling axis could prove to be a viable treatment option for a variety of neurodegenerative diseases.

## Conclusions and Future Directions

**Figure 3:**
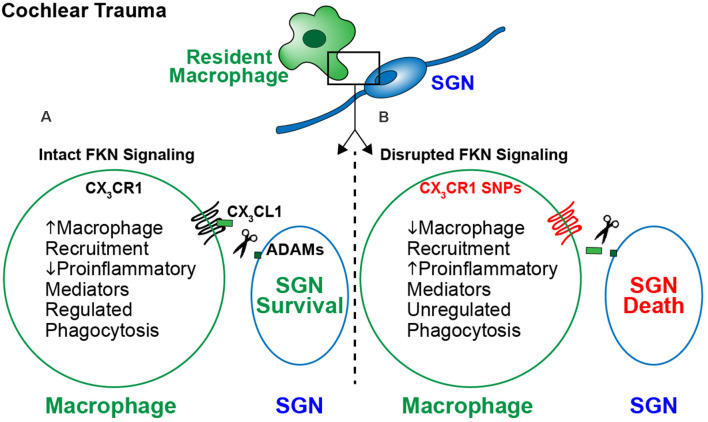
The proposed model for fractalkine-mediated neuroprotection after cochlear injury. Following cochlear damage there is a sustained increase in the numbers of macrophages in the spiral ganglion. This increase in numbers is likely due to infiltration of blood-derived macrophages from the vasculature. **(A)** Following cochlear trauma, CX_3_CL1 is proteolytically cleaved from SGNs/IHCs and soluble CX_3_CL1 binds to its unique receptor CX_3_CR1 expressed by macrophages. Activation of fractalkine signaling induces an immune response characterized by recruitment of anti-inflammatory and pro-healing macrophages from the vasculature into the injured cochlea, regulation of cochlear inflammation, and preserved SGNs. **(B)** When FKN signaling is disrupted, i.e., due to loss of function in humans carrying CX3CR1 polymorphisms, there is a lack of macrophage recruitment and a sustained proinflammatory state following trauma. This results in SGN death, due to unregulated inflammation and phagocytosis by macrophages.

The study of immune dysfunction continues to accumulate evidence for a primary role in the development and progression of neurodegenerative diseases. Specifically, interest in immune signaling pathways has grown, due to their involvement in tissue development, tissue homeostasis, and immune surveillance. Dysfunction in these communication pathways can result in neurotoxicity, resulting from unregulated immune activation. Evidence in several neurodegenerative diseases, including AD, PD, MS, and diseases of the retina suggests FKN signaling is responsible for dampening microglia activation, resulting in neuroprotection. Emerging research also suggests FKN signaling plays an important role in inner ear neuroprotection. However, the question remains, what is the specific role of macrophages and FKN signaling in cochlear pathology as well as other neurological conditions? We hypothesize that FKN signaling plays dual roles during cochlear pathology, as a chemokine and a checkpoint molecule. As a chemokine, FKN signaling mediates cochlear sensory and neuron protection by recruitment of anti-inflammatory and pro-healing macrophages from the circulation to the site of damage, which may be context or injury dependent. As a check point molecule, FKN signaling modulates the cochlear inflammatory state by regulating the inflammatory and phagocytic phenotype of resident and circulating macrophages. These functions may be inclusive or exclusive (Figure 3). However, if FKN signaling is disrupted, which in humans can occur through the previously mentioned CX_3_CR1 SNPs, there is diminished recruitment of peripheral macrophages and dysregulated production of proinflammatory mediators, leading to unregulated phagocytosis and SGN death. If accurate, these hypotheses would support the viability of exogenous FKN as a therapeutic mechanism to treat inner ear pathology. However, to fully determine the role of FKN signaling in cochlear physiology and pathology, the following questions need to be carefully addressed:

1. What is the role of the FKN ligand in normal and damaged cochlea? To this point, FKN’s role in modulating cochlear pathology has only been investigated in terms of the presence or absence of its cognate receptor CX_3_CR1. Therefore, determining the effects of presence and absence of FKN ligand in the normal and damaged cochlea and in oto- and neuroprotection and the factors that regulate the endogenous levels of soluble and membrane-bound FKN in the injured cochlea are equally vital.
2. What is the precise role of CX_3_CR1 expressing resident and recruited macrophages in SGN survival? CX_3_CR1 is expressed on both cochlear resident macrophages and on macrophages that migrate to the damaged cochlea from blood circulation. However, to what extent CX_3_CR1-expressing resident and/or recruited macrophages contribute towards the long-term survival of SGNs in the damaged cochlea remains to be determined.
3. Is FKN a chemotactic signal for resident or recruited macrophages in the injured cochlea? The FKN signaling has been explored elsewhere in the body for its role in chemotactic signaling to recruit additional immune cells to sites of tissue damage. Following cochlear trauma, there is an infiltration of circulating monocyte-derived macrophages to the site of injury. However, it is unclear if the FKN signaling axis is responsible for this migration of macrophages to the cochlea. Additionally, it is unclear if within the cochlea, FKN signaling acts as a chemotactic signal to draw additional resident macrophages from other regions of the cochlea to the site of cellular damage.
4. Does CX_3_CR1 regulate cochlear inflammation and immune response to promote SGN survival following injury? It is not known if FKN signaling is directly responsible for protecting SGNs following trauma, and furthermore, if this neuroprotection is related to its regulation of proinflammatory mediator production and macrophage phagocytosis in the injured cochlea.
5. What is the association of human CX_3_CR1 SNPs in acquired SNHL? Studies have shown a causal link between human CX_3_CR1 SNPs and increased susceptibility to various neurodegenerative diseases. However, it remains unknown if these SNPs are also associated with the development, progression, or severity of cochlear pathology and hearing loss in humans.
6. Can FKN overexpression or increase in CX_3_CR1 activity be used as a viable therapeutic target for SGN survival and ribbon synapse regeneration? Based on the number of studies indicated above on the therapeutic viability of using AAV or peptides to overexpress soluble FKN for the treatment of neurodegenerative diseases, it will be of great interest to examine the effects of overexpression of FKN as a viable treatment option for the survival of SGNs and regeneration of damaged ribbon synapses following cochlear trauma. Such putative therapeutics options may be a viable treatment for hidden-hearing loss.

Addressing the above questions, we will better understand the role FKN signaling has on immune regulation in the inner ear during cochlear trauma. By doing so, we can begin to develop therapeutic targets for not only prophylactic protection but post-trauma recovery. Together, understanding the role of FKN signaling in inner ear pathology has great promise for unraveling the mysteries tied to the treatment of hearing loss.

## Author Contributions

AS reviewed the literature and wrote the manuscript. TK wrote and edited the manuscript. All authors contributed to the article and approved the submitted version.

## Conflict of Interest

The authors declare that the research was conducted in the absence of any commercial or financial relationships that could be construed as a potential conflict of interest.

## Publisher’s Note

All claims expressed in this article are solely those of the authors and do not necessarily represent those of their affiliated organizations, or those of the publisher, the editors and the reviewers. Any product that may be evaluated in this article, or claim that may be made by its manufacturer, is not guaranteed or endorsed by the publisher.

## References

[B1] Aguzzi A. O’Connor T. (2010). Protein aggregation diseases: pathogenicity and therapeutic perspectives. Nat. Rev. Drug. Discov. 9, 237–248. 10.1038/nrd3050 20190788

[B2] Almasieh M. Wilson A. M. Morquette B. Cueva Vargas J. L. Di Polo A. (2012). The molecular basis of retinal ganglion cell death in glaucoma. Prog. Retin. Eye Res. 31, 152–181. 10.1016/j.preteyeres.2011.11.002 22155051

[B3] Ambati J. Anand A. Fernandez S. Sakurai E. Lynn B. C. Kuziel W. A.. (2003). An animal model of age-related macular degeneration in senescent Ccl-2- or Ccr-2-deficient mice. Nat. Med. 9, 1390–1397. 10.1038/nm950 14566334

[B4] Antonetti D. A. Klein R. Gardner T. W. (2012). Diabetic retinopathy. N. Engl. J. Med. 366, 1227–1239. 10.1056/NEJMra1005073 22455417

[B5] Arango Duque G. Descoteaux A. (2014). Macrophage cytokines: involvement in immunity and infectious diseases. Front. Immunol. 5:491. 10.3389/fimmu.2014.00491 25339958 PMC4188125

[B6] Barber A. J. (2003). A new view of diabetic retinopathy: a neurodegenerative disease of the eye. Prog. Neuropsychopharmacol. Biol. Psychiatry 27, 283–290. 10.1016/S0278-5846(03)00023-X 12657367

[B7] Bazan J. F. Bacon K. B. Hardiman G. Wang W. Soo K. Rossi D.. (1997). A new class of membrane-bound chemokine with a CX3C motif. Nature 385, 640–644. 10.1038/385640a0 9024663

[B8] Biber K. Neumann H. Inoue K. Boddeke H. W. (2007). Neuronal “on” and “off” signals control microglia. Trends Neurosci. 30, 596–602. 10.1016/j.tins.2007.08.007 17950926

[B9] Bolos M. Llorens-Martin M. Perea J. R. Jurado-Arjona J. Rabano A. Hernandez F.. (2017). Absence of CX3CR1 impairs the internalization of Tau by microglia. Mol. Neurodegener. 12:59. 10.1186/s13024-017-0200-1 28810892 PMC5558740

[B10] Bosco A. Steele M. R. Vetter M. L. (2011). Early microglia activation in a mouse model of chronic glaucoma. J. Comp. Neurol. 519, 599–620. 10.1002/cne.22516 21246546 PMC4169989

[B11] Cao Q. Wang Y. Wang X. M. Lu J. Lee V. W. Ye Q.. (2015). Renal F4/80+ CD11c+ mononuclear phagocytes display phenotypic and functional characteristics of macrophages in health and in adriamycin nephropathy. J. Am. Soc. Nephrol. 26, 349–363. 10.1681/ASN.2013121336 25012165 PMC4310657

[B12] Cardona A. E. Pioro E. P. Sasse M. E. Kostenko V. Cardona S. M. Dijkstra I. M.. (2006). Control of microglial neurotoxicity by the fractalkine receptor. Nat. Neurosci. 9, 917–924. 10.1038/nn1715 16732273

[B13] Cardona S. M. Kim S. V. Church K. A. Torres V. O. Cleary I. A. Mendiola A. S.. (2018). Role of the fractalkine receptor in CNS autoimmune inflammation: new approach utilizing a mouse model expressing the human CX3CR1(I249/M280) variant. Front. Cell. Neurosci. 12:365. 10.3389/fncel.2018.00365 30386211 PMC6199958

[B14] Cardona S. M. Mendiola A. S. Yang Y. C. Adkins S. L. Torres V. Cardona A. E.. (2015). Disruption of fractalkine signaling leads to microglial activation and neuronal damage in the diabetic retina. ASN Neuro 7:1759091415608204. 10.1177/1759091415608204 26514658 PMC4641555

[B15] Catalano M. Lauro C. Cipriani R. Chece G. Ponzetta A. Di Angelantonio S.. (2013). CX3CL1 protects neurons against excitotoxicity enhancing GLT-1 activity on astrocytes. J. Neuroimmunol. 263, 75–82. 10.1016/j.jneuroim.2013.07.020 23968561

[B16] Chan C. C. Tuo J. Bojanowski C. M. Csaky K. G. Green W. R. (2005). Detection of CX3CR1 single nucleotide polymorphism and expression on archived eyes with age-related macular degeneration. Histol. Histopathol. 20, 857–863. 10.14670/HH-20.857 15944936 PMC1930145

[B17] Chaplin D. D. (2010). Overview of the immune response. J. Allergy Clin. Immunol. 125, S3–S23. 10.1016/j.jaci.2009.12.980 20176265 PMC2923430

[B18] Chapman G. A. Moores K. Harrison D. Campbell C. A. Stewart B. R. Strijbos P. J.. (2000). Fractalkine cleavage from neuronal membranes represents an acute event in the inflammatory response to excitotoxic brain damage. J. Neurosci. 20:RC87. 10.1523/JNEUROSCI.20-15-j0004.2000 10899174 PMC6772533

[B19] Cho S. H. Sun B. Zhou Y. Kauppinen T. M. Halabisky B. Wes P.. (2011). CX3CR1 protein signaling modulates microglial activation and protects against plaque-independent cognitive deficits in a mouse model of Alzheimer disease. J. Biol. Chem. 286, 32713–32722. 10.1074/jbc.M111.254268 21771791 PMC3173153

[B20] Clark A. K. Malcangio M. (2012). Microglial signalling mechanisms: cathepsin S and fractalkine. Exp. Neurol. 234, 283–292. 10.1016/j.expneurol.2011.09.012 21946268

[B21] Coate T. M. Scott M. K. Gurjar M. (2019). Current concepts in cochlear ribbon synapse formation. Synapse 73:e22087. 10.1002/syn.22087 30592086 PMC6573016

[B22] Colonna M. Butovsky O. (2017). Microglia function in the central nervous system during health and neurodegeneration. Annu. Rev. Immunol. 35, 441–468. 10.1146/annurev-immunol-051116-052358 28226226 PMC8167938

[B23] Cooper M. D. Alder M. N. (2006). The evolution of adaptive immune systems. Cell 124, 815–822. 10.1016/j.cell.2006.02.001 16497590

[B24] Cui Q. Yin Y. Benowitz L. I. (2009). The role of macrophages in optic nerve regeneration. Neuroscience 158, 1039–1048. 10.1016/j.neuroscience.2008.07.036 18708126 PMC2670061

[B25] Dalby N. O. Mody I. (2001). The process of epileptogenesis: a pathophysiological approach. Curr. Opin. Neurol. 14, 187–192. 10.1097/00019052-200104000-00009 11262734

[B26] Dantzer R. (2018). Neuroimmune interactions: from the brain to the immune system and vice versa. Physiol. Rev. 98, 477–504. 10.1152/physrev.00039.2016 29351513 PMC5866360

[B27] Deiva K. Geeraerts T. Salim H. Leclerc P. Hery C. Hugel B.. (2004). Fractalkine reduces N-methyl-d-aspartate-induced calcium flux and apoptosis in human neurons through extracellular signal-regulated kinase activation. Eur. J. Neurosci. 20, 3222–3232. 10.1111/j.1460-9568.2004.03800.x 15610155

[B28] Dong Y. Zhang C. Frye M. Yang W. Ding D. Sharma A.. (2018). Differential fates of tissue macrophages in the cochlea during postnatal development. Hear. Res. 365, 110–126. 10.1016/j.heares.2018.05.010 29804721 PMC6026078

[B29] Dupont J. Guilhaume A. Aran J. M. (1993). Neuronal degeneration of primary cochlear and vestibular innervations after local injection of sisomicin in the guinea pig. Hear. Res. 68, 217–228. 10.1016/0378-5955(93)90125-k 8407607

[B30] Ebneter A. Casson R. J. Wood J. P. Chidlow G. (2010). Microglial activation in the visual pathway in experimental glaucoma: spatiotemporal characterization and correlation with axonal injury. Invest. Ophthalmol. Vis. Sci. 51, 6448–6460. 10.1167/iovs.10-5284 20688732

[B31] Echteler S. M. Magardino T. Rontal M. (2005). Spatiotemporal patterns of neuronal programmed cell death during postnatal development of the gerbil cochlea. Brain Res. Dev. Brain Res. 157, 192–200. 10.1016/j.devbrainres.2005.04.004 15939482

[B32] Elliott M. R. Koster K. M. Murphy P. S. (2017). Efferocytosis signaling in the regulation of macrophage inflammatory responses. J. Immunol. 198, 1387–1394. 10.4049/jimmunol.1601520 28167649 PMC5301545

[B33] Epelman S. Lavine K. J. Randolph G. J. (2014). Origin and functions of tissue macrophages. Immunity 41, 21–35. 10.1016/j.immuni.2014.06.013 25035951 PMC4470379

[B34] Eyo U. B. Murugan M. Wu L. J. (2017). Microglia-neuron communication in epilepsy. Glia 65, 5–18. 10.1002/glia.23006 27189853 PMC5116010

[B35] Eyo U. B. Peng J. Murugan M. Mo M. Lalani A. Xie P.. (2016). Regulation of physical microglia-neuron interactions by fractalkine signaling after status epilepticus. eNeuro 3:ENEURO.0209-16.2016. 10.1523/ENEURO.0209-16.2016 28101527 PMC5237828

[B36] Faure S. Meyer L. Costagliola D. Vaneensberghe C. Genin E. Autran B.. (2000). Rapid progression to AIDS in HIV+ individuals with a structural variant of the chemokine receptor CX3CR1. Science 287, 2274–2277. 10.1126/science.287.5461.2274 10731151

[B37] Finneran D. J. Nash K. R. (2019). Neuroinflammation and fractalkine signaling in Alzheimer’s disease. J. Neuroinflammation 16:30. 10.1186/s12974-019-1412-9 30744705 PMC6371521

[B38] Finneran D. J. Morgan D. Gordon M. N. Nash K. R. (2019). CNS-wide over expression of fractalkine improves cognitive functioning in a tauopathy model. J. Neuroimmune Pharmacol. 14, 312–325. 10.1007/s11481-018-9822-5 30499006 PMC6525127

[B39] Fisher R. S. van Emde Boas W. Blume W. Elger C. Genton P. Lee P.. (2005). Epileptic seizures and epilepsy: definitions proposed by the international league against epilepsy (ILAE) and the international bureau for epilepsy (IBE). Epilepsia 46, 470–472. 10.1111/j.0013-9580.2005.66104.x 15816939

[B40] Fredelius L. Rask-Andersen H. (1990). The role of macrophages in the disposal of degeneration products within the organ of corti after acoustic overstimulation. Acta Otolaryngol. 109, 76–82. 10.3109/00016489009107417 2309562

[B41] Frodermann V. Nahrendorf M. (2018). Macrophages and cardiovascular health. Physiol. Rev. 98, 2523–2569. 10.1152/physrev.00068.2017 30156496 PMC6442921

[B42] Frye M. D. Yang W. Zhang C. Xiong B. Hu B. H. (2017). Dynamic activation of basilar membrane macrophages in response to chronic sensory cell degeneration in aging mouse cochleae. Hear. Res. 344, 125–134. 10.1016/j.heares.2016.11.003 27837652 PMC5239751

[B43] Fujioka M. Okano H. Ogawa K. (2014). Inflammatory and immune responses in the cochlea: potential therapeutic targets for sensorineural hearing loss. Front. Pharmacol. 5:287. 10.3389/fphar.2014.00287 25566079 PMC4274906

[B44] Garcia J. A. Pino P. A. Mizutani M. Cardona S. M. Charo I. F. Ransohoff R. M.. (2013). Regulation of adaptive immunity by the fractalkine receptor during autoimmune inflammation. J. Immunol. 191, 1063–1072. 10.4049/jimmunol.1300040 23817416 PMC3720756

[B45] Geissmann F. Jung S. Littman D. R. (2003). Blood monocytes consist of two principal subsets with distinct migratory properties. Immunity 19, 71–82. 10.1016/s1074-7613(03)00174-2 12871640

[B46] Gillespie L. N. Shepherd R. K. (2005). Clinical application of neurotrophic factors: the potential for primary auditory neuron protection. Eur. J. Neurosci. 22, 2123–2133. 10.1111/j.1460-9568.2005.04430.x 16262651 PMC1831824

[B47] Ginhoux F. Guilliams M. (2016). Tissue-resident macrophage ontogeny and homeostasis. Immunity 44, 439–449. 10.1016/j.immuni.2016.02.024 26982352

[B48] Ginhoux F. Greter M. Leboeuf M. Nandi S. See P. Gokhan S.. (2010). Fate mapping analysis reveals that adult microglia derive from primitive macrophages. Science 330, 841–845. 10.1126/science.1194637 20966214 PMC3719181

[B49] Golub J. S. Tong L. Ngyuen T. B. Hume C. R. Palmiter R. D. Rubel E. W.. (2012). Hair cell replacement in adult mouse utricles after targeted ablation of hair cells with diphtheria toxin. J. Neurosci. 32, 15093–15105. 10.1523/JNEUROSCI.1709-12.2012 23100430 PMC3544304

[B50] Gomez Perdiguero E. Klapproth K. Schulz C. Busch K. Azzoni E. Crozet L.. (2015). Tissue-resident macrophages originate from yolk-sac-derived erythro-myeloid progenitors. Nature 518, 547–551. 10.1038/nature13989 25470051 PMC5997177

[B51] Gordon S. Martinez-Pomares L. (2017). Physiological roles of macrophages. Pflugers Arch. 469, 365–374. 10.1007/s00424-017-1945-7 28185068 PMC5362657

[B52] Gordon S. Pluddemann A. (2017). Tissue macrophages: heterogeneity and functions. BMC Biol. 15:53. 10.1186/s12915-017-0392-4 28662662 PMC5492929

[B53] Hammond T. R. Dufort C. Dissing-Olesen L. Giera S. Young A. Wysoker A.. (2019). Single-cell RNA sequencing of microglia throughout the mouse lifespan and in the injured brain reveals complex cell-state changes. Immunity 50, 253.e6–271.e6. 10.1016/j.immuni.2018.11.004 30471926 PMC6655561

[B54] Harris J. P. (1983). Immunology of the inner ear: response of the inner ear to antigen challenge. Otolaryngol. Head Neck Surg. 91, 18–32. 10.1177/019459988309100105 6405344

[B55] Harris J. P. (1984). Immunology of the inner ear: evidence of local antibody production. Ann. Otol. Rhinol. Laryngol. 93, 157–162. 10.1177/000348948409300211 6712089

[B56] Harrison J. K. Jiang Y. Chen S. Xia Y. Maciejewski D. McNamara R. K.. (1998). Role for neuronally derived fractalkine in mediating interactions between neurons and CX3CR1-expressing microglia. Proc. Natl. Acad. Sci. U S A 95, 10896–10901. 10.1073/pnas.95.18.10896 9724801 PMC27992

[B57] Hartong D. T. Berson E. L. Dryja T. P. (2006). Retinitis pigmentosa. Lancet 368, 1795–1809. 10.1016/S0140-6736(06)69740-7 17113430

[B58] Hashimoto D. Chow A. Noizat C. Teo P. Beasley M. B. Leboeuf M.. (2013). Tissue-resident macrophages self-maintain locally throughout adult life with minimal contribution from circulating monocytes. Immunity 38, 792–804. 10.1016/j.immuni.2013.04.004 23601688 PMC3853406

[B59] Haskell C. A. Cleary M. D. Charo I. F. (1999). Molecular uncoupling of fractalkine-mediated cell adhesion and signal transduction. Rapid flow arrest of CX3CR1-expressing cells is independent of G-protein activation. J. Biol. Chem. 274, 10053–10058. 10.1074/jbc.274.15.10053 10187784

[B60] He W. Yu J. Sun Y. Kong W. (2020). Macrophages in noise-exposed cochlea: changes, regulation and the potential role. Aging Dis. 11, 191–199. 10.14336/AD.2019.0723 32010492 PMC6961779

[B61] Heneka M. T. Kummer M. P. Latz E. (2014). Innate immune activation in neurodegenerative disease. Nat. Rev. Immunol. 14, 463–477. 10.1038/nri3705 24962261

[B62] Hermand P. Pincet F. Carvalho S. Ansanay H. Trinquet E. Daoudi M.. (2008). Functional adhesiveness of the CX3CL1 chemokine requires its aggregation. Role of the transmembrane domain. J. Biol. Chem. 283, 30225–30234. 10.1074/jbc.M802638200 18725411 PMC2662081

[B63] Hirayama D. Iida T. Nakase H. (2017). The phagocytic function of macrophage-enforcing innate immunity and tissue homeostasis. Int. J. Mol. Sci. 19:92. 10.3390/ijms19010092 29286292 PMC5796042

[B64] Hirose K. Sato E. (2011). Comparative analysis of combination kanamycin-furosemide versus kanamycin alone in the mouse cochlea. Hear. Res. 272, 108–116. 10.1016/j.heares.2010.10.011 21044672 PMC4519356

[B65] Hirose K. Discolo C. M. Keasler J. R. Ransohoff R. (2005). Mononuclear phagocytes migrate into the murine cochlea after acoustic trauma. J. Comp. Neurol. 489, 180–194. 10.1002/cne.20619 15983998

[B66] Hirose K. Li S. Z. Ohlemiller K. K. Ransohoff R. M. (2014). Systemic lipopolysaccharide induces cochlear inflammation and exacerbates the synergistic ototoxicity of kanamycin and furosemide. J. Assoc. Res. Otolaryngol. 15, 555–570. 10.1007/s10162-014-0458-8 24845404 PMC4141430

[B67] Hoeffel G. Wang Y. Greter M. See P. Teo P. Malleret B.. (2012). Adult Langerhans cells derive predominantly from embryonic fetal liver monocytes with a minor contribution of yolk sac-derived macrophages. J. Exp. Med. 209, 1167–1181. 10.1084/jem.20120340 22565823 PMC3371735

[B68] Hoshiko M. Arnoux I. Avignone E. Yamamoto N. Audinat E. (2012). Deficiency of the microglial receptor CX3CR1 impairs postnatal functional development of thalamocortical synapses in the barrel cortex. J. Neurosci. 32, 15106–15111. 10.1523/JNEUROSCI.1167-12.2012 23100431 PMC6704837

[B69] Hua J. Y. Smith S. J. (2004). Neural activity and the dynamics of central nervous system development. Nat. Neurosci. 7, 327–332. 10.1038/nn1218 15048120

[B70] Hughes C. E. Nibbs R. J. B. (2018). A guide to chemokines and their receptors. FEBS J. 285, 2944–2971. 10.1111/febs.14466 29637711 PMC6120486

[B71] Hundhausen C. Misztela D. Berkhout T. A. Broadway N. Saftig P. Reiss K.. (2003). The disintegrin-like metalloproteinase ADAM10 is involved in constitutive cleavage of CX3CL1 (fractalkine) and regulates CX3CL1-mediated cell-cell adhesion. Blood 102, 1186–1195. 10.1182/blood-2002-12-3775 12714508

[B72] Imai T. Hieshima K. Haskell C. Baba M. Nagira M. Nishimura M.. (1997). Identification and molecular characterization of fractalkine receptor CX3CR1, which mediates both leukocyte migration and adhesion. Cell 91, 521–530. 10.1016/s0092-8674(00)80438-9 9390561

[B73] Infante-Duarte C. Weber A. Kratzschmar J. Prozorovski T. Pikol S. Hamann I.. (2005). Frequency of blood CX3CR1-positive natural killer cells correlates with disease activity in multiple sclerosis patients. FASEB J. 19, 1902–1904. 10.1096/fj.05-3832fje 16144955

[B74] International Multiple Sclerosis Genetics Consortium and the Wellcome Trust Case Control Consortium Wellcome Trust Case Control Sawcer S. Hellenthal G. Pirinen M. Spencer C. C.. (2011). Genetic risk and a primary role for cell-mediated immune mechanisms in multiple sclerosis. Nature 476, 214–219. 10.1038/nature10251 21833088 PMC3182531

[B75] Ising C. Heneka M. T. (2018). Functional and structural damage of neurons by innate immune mechanisms during neurodegeneration. Cell Death Dis. 9:120. 10.1038/s41419-017-0153-x 29371603 PMC5833757

[B76] Jones B. A. Beamer M. Ahmed S. (2010). Fractalkine/CX3CL1: a potential new target for inflammatory diseases. Mol. Interv. 10, 263–270. 10.1124/mi.10.5.3 21045240 PMC3002219

[B77] Jones B. A. Riegsecker S. Rahman A. Beamer M. Aboualaiwi W. Khuder S. A.. (2013). Role of ADAM-17, p38 MAPK, cathepsins and the proteasome pathway in the synthesis and shedding of fractalkine/CX(3) CL1 in rheumatoid arthritis. Arthritis Rheum. 65, 2814–2825. 10.1002/art.38095 23897050

[B78] Julia V. (2012). CX3CL1 in allergic diseases: not just a chemotactic molecule. Allergy 67, 1106–1110. 10.1111/j.1398-9995.2012.02870.x 22765026

[B79] Jung S. Aliberti J. Graemmel P. Sunshine M. J. Kreutzberg G. W. Sher A.. (2000). Analysis of fractalkine receptor CX(3)CR1 function by targeted deletion and green fluorescent protein reporter gene insertion. Mol. Cell Biol. 20, 4106–4114. 10.1128/MCB.20.11.4106-4114.2000 10805752 PMC85780

[B80] Kakinuma T. Hwang S. T. (2006). Chemokines, chemokine receptors and cancer metastasis. J. Leukoc. Biol. 79, 639–651. 10.1189/jlb.1105633 16478915

[B81] Kato G. Inada H. Wake H. Akiyoshi R. Miyamoto A. Eto K.. (2016). Microglial contact prevents excess depolarization and rescues neurons from excitotoxicity. eNeuro 3:ENEURO.0004-16.2016. 10.1523/ENEURO.0004-16.2016 27390772 PMC4916329

[B82] Kaur T. Clayman A. C. Nash A. J. Schrader A. D. Warchol M. E. Ohlemiller K. K. (2019). Lack of fractalkine receptor on macrophages impairs spontaneous recovery of ribbon synapses after moderate noise trauma in C57BL/6 Mice. Front. Neurosci. 13:620. 10.3389/fnins.2019.00620 31263398 PMC6585312

[B83] Kaur T. Ohlemiller K. K. Warchol M. E. (2018). Genetic disruption of fractalkine signaling leads to enhanced loss of cochlear afferents following ototoxic or acoustic injury. J. Comp. Neurol. 526, 824–835. 10.1002/cne.24369 29218724 PMC5903687

[B84] Kaur T. Zamani D. Tong L. Rubel E. W. Ohlemiller K. K. Hirose K.. (2015). Fractalkine signaling regulates macrophage recruitment into the cochlea and promotes the survival of spiral ganglion neurons after selective hair cell lesion. J. Neurosci. 35, 15050–15061. 10.1523/JNEUROSCI.2325-15.2015 26558776 PMC4642237

[B85] Kerfoot S. M. Lord S. E. Bell R. B. Gill V. Robbins S. M. Kubes P. (2003). Human fractalkine mediates leukocyte adhesion but not capture under physiological shear conditions; a mechanism for selective monocyte recruitment. Eur. J. Immunol. 33, 729–739. 10.1002/eji.200323502 12616493

[B86] Kishimoto I. Okano T. Nishimura K. Motohashi T. Omori K. (2019). Early development of resident macrophages in the mouse cochlea depends on yolk sac hematopoiesis. Front. Neurol. 10:1115. 10.3389/fneur.2019.01115 31695671 PMC6817595

[B87] Klosowska K. Volin M. V. Huynh N. Chong K. K. Halloran M. M. Woods J. M. (2009). Fractalkine functions as a chemoattractant for osteoarthritis synovial fibroblasts and stimulates phosphorylation of mitogen-activated protein kinases and Akt. Clin. Exp. Immunol. 156, 312–319. 10.1111/j.1365-2249.2009.03903.x 19302240 PMC2759480

[B88] Koch A. E. (2005). Chemokines and their receptors in rheumatoid arthritis: future targets? Arthritis Rheum. 52, 710–721. 10.1002/art.20932 15751074

[B89] Kujawa S. G. Liberman M. C. (2009). Adding insult to injury: cochlear nerve degeneration after "temporary" noise-induced hearing loss. J. Neurosci. 29, 14077–14085. 10.1523/JNEUROSCI.2845-09.2009 19906956 PMC2812055

[B90] Lang H. Ebihara Y. Schmiedt R. A. Minamiguchi H. Zhou D. Smythe N.. (2006). Contribution of bone marrow hematopoietic stem cells to adult mouse inner ear: mesenchymal cells and fibrocytes. J. Comp. Neurol. 496, 187–201. 10.1002/cne.20929 16538683 PMC2561311

[B91] Lang H. Nishimoto E. Xing Y. Brown L. N. Noble K. V. Barth J. L.. (2016). Contributions of mouse and human hematopoietic cells to remodeling of the adult auditory nerve after neuron loss. Mol. Ther. 24, 2000–2011. 10.1038/mt.2016.174 27600399 PMC5154482

[B92] Lauro C. (2015). Fractalkine: multiple strategies to counteract glutamate receptors activation leading to neuroprotection. Neural Regen. Res. 10, 1214–1215. 10.4103/1673-5374.162697 26487840 PMC4590225

[B93] Lee M. Lee Y. Song J. Lee J. Chang S. Y. (2018). Tissue-specific Role of CX3CR1 expressing immune cells and their relationships with human disease. Immune Netw. 18:e5. 10.4110/in.2018.18.e5 29503738 PMC5833124

[B94] Lee S. Xu G. Jay T. R. Bhatta S. Kim K. W. Jung S.. (2014). Opposing effects of membrane-anchored CX3CL1 on amyloid and tau pathologies via the p38 MAPK pathway. J. Neurosci. 34, 12538–12546. 10.1523/JNEUROSCI.0853-14.2014 25209291 PMC4160782

[B95] Liang K. J. Lee J. E. Wang Y. D. Ma W. Fontainhas A. M. Fariss R. N.. (2009). Regulation of dynamic behavior of retinal microglia by CX3CR1 signaling. Invest. Ophthalmol. Vis. Sci. 50, 4444–4451. 10.1167/iovs.08-3357 19443728 PMC2749316

[B96] Liberman M. C. (1984). Single-neuron labeling and chronic cochlear pathology. I. Threshold shift and characteristic-frequency shift. Hear. Res. 16, 33–41. 10.1016/0378-5955(84)90023-6 6096345

[B97] Liberman M. C. Kujawa S. G. (2017). Cochlear synaptopathy in acquired sensorineural hearing loss: Manifestations and mechanisms. Hear. Res. 349, 138–147. 10.1016/j.heares.2017.01.003 28087419 PMC5438769

[B98] Limatola C. Lauro C. Catalano M. Ciotti M. T. Bertollini C. Di Angelantonio S.. (2005). Chemokine CX3CL1 protects rat hippocampal neurons against glutamate-mediated excitotoxicity. J. Neuroimmunol. 166, 19–28. 10.1016/j.jneuroim.2005.03.023 16019082

[B99] Liu W. Molnar M. Garnham C. Benav H. Rask-Andersen H. (2018). Macrophages in the human cochlea: saviors or predators-a study using super-resolution immunohistochemistry. Front. Immunol. 9:223. 10.3389/fimmu.2018.00223 29487598 PMC5816790

[B100] Liu Z. Condello C. Schain A. Harb R. Grutzendler J. (2010). CX3CR1 in microglia regulates brain amyloid deposition through selective protofibrillar amyloid-beta phagocytosis. J. Neurosci. 30, 17091–17101. 10.1523/JNEUROSCI.4403-10.2010 21159979 PMC3077120

[B101] Lucin K. M. Wyss-Coray T. (2009). Immune activation in brain aging and neurodegeneration: too much or too little? Neuron 64, 110–122. 10.1016/j.neuron.2009.08.039 19840553 PMC2834890

[B102] Ma B. Dang G. Yang S. Duan L. Zhang Y. (2015). CX3CR1 polymorphisms and the risk of age-related macular degeneration. Int. J. Clin. Exp. Pathol. 8, 9592–9596. 26464724 PMC4583956

[B103] Maggi L. Scianni M. Branchi I. D’Andrea I. Lauro C. Limatola C. (2011). CX(3)CR1 deficiency alters hippocampal-dependent plasticity phenomena blunting the effects of enriched environment. Front. Cell. Neurosci. 5:22. 10.3389/fncel.2011.00022 22025910 PMC3198035

[B104] Marinelli S. Basilico B. Marrone M. C. Ragozzino D. (2019). Microglia-neuron crosstalk: signaling mechanism and control of synaptic transmission. Semin. Cell Dev. Biol. 94, 138–151. 10.1016/j.semcdb.2019.05.017 31112798

[B105] McCabe B. F. (1989). Autoimmune inner ear disease: therapy. Am. J. Otol. 10, 196–197. 2750868

[B106] McDermott D. H. Fong A. M. Yang Q. Sechler J. M. Cupples L. A. Merrell M. N.. (2003). Chemokine receptor mutant CX3CR1–M280 has impaired adhesive function and correlates with protection from cardiovascular disease in humans. J. Clin. Invest. 111, 1241–1250. 10.1172/JCI16790 12697743 PMC152935

[B107] Meghraoui-Kheddar A. Barthelemy S. Boissonnas A. Combadiere C. (2020). Revising CX3CR1 expression on murine classical and non-classical monocytes. Front. Immunol. 11:1117. 10.3389/fimmu.2020.01117 32582197 PMC7283740

[B108] Mendiola A. S. Garza R. Cardona S. M. Mythen S. A. Lira S. A. Akassoglou K.. (2016). Fractalkine signaling attenuates perivascular clustering of microglia and fibrinogen leakage during systemic inflammation in mouse models of diabetic retinopathy. Front. Cell. Neurosci. 10:303. 10.3389/fncel.2016.00303 28119571 PMC5222852

[B109] Mollica Poeta V. Massara M. Capucetti A. Bonecchi R. (2019). Chemokines and chemokine receptors: new targets for cancer immunotherapy. Front. Immunol. 10:379. 10.3389/fimmu.2019.00379 30894861 PMC6414456

[B110] Morganti J. M. Nash K. R. Grimmig B. A. Ranjit S. Small B. Bickford P. C.. (2012). The soluble isoform of CX3CL1 is necessary for neuroprotection in a mouse model of Parkinson’s disease. J. Neurosci. 32, 14592–14601. 10.1523/JNEUROSCI.0539-12.2012 23077045 PMC3501652

[B111] Mosser D. M. Hamidzadeh K. Goncalves R. (2021). Macrophages and the maintenance of homeostasis. Cell Mol. Immunol. 18, 579–587. 10.1038/s41423-020-00541-3 32934339 PMC7491045

[B112] Nash K. R. Lee D. C. Hunt J. B. Jr. Morganti J. M. Selenica M. L. Moran P.. (2013). Fractalkine overexpression suppresses tau pathology in a mouse model of tauopathy. Neurobiol. Aging 34, 1540–1548. 10.1016/j.neurobiolaging.2012.12.011 23332170 PMC8970215

[B113] Nash K. R. Moran P. Finneran D. J. Hudson C. Robinson J. Morgan D.. (2015). Fractalkine over expression suppresses alpha-synuclein-mediated neurodegeneration. Mol. Ther. 23, 17–23. 10.1038/mt.2014.175 25195598 PMC4426798

[B114] Nickells R. W. Howell G. R. Soto I. John S. W. (2012). Under pressure: cellular and molecular responses during glaucoma, a common neurodegeneration with axonopathy. Annu. Rev. Neurosci. 35, 153–179. 10.1146/annurev.neuro.051508.135728 22524788

[B115] Nimmerjahn A. Kirchhoff F. Helmchen F. (2005). Resting microglial cells are highly dynamic surveillants of brain parenchyma *in vivo*. Science 308, 1314–1318. 10.1126/science.1110647 15831717

[B116] Noble K. V. Liu T. Matthews L. J. Schulte B. A. Lang H. (2019). Age-related changes in immune cells of the human cochlea. Front. Neurol. 10:895. 10.3389/fneur.2019.00895 31474935 PMC6707808

[B117] Noda M. Doi Y. Liang J. Kawanokuchi J. Sonobe Y. Takeuchi H.. (2011). Fractalkine attenuates excito-neurotoxicity via microglial clearance of damaged neurons and antioxidant enzyme heme oxygenase-1 expression. J. Biol. Chem. 286, 2308–2319. 10.1074/jbc.M110.169839 21071446 PMC3023525

[B118] Oesterle E. C. Campbell S. Taylor R. R. Forge A. Hume C. R. (2008). Sox2 and JAGGED1 expression in normal and drug-damaged adult mouse inner ear. J. Assoc. Res. Otolaryngol. 9, 65–89. 10.1007/s10162-007-0106-7 18157569 PMC2536811

[B119] Okano T. Kishimoto I. (2019). Csf1 signaling regulates maintenance of resident macrophages and bone formation in the mouse cochlea. Front. Neurol. 10:1244. 10.3389/fneur.2019.01244 31824413 PMC6881377

[B120] Okano T. Nakagawa T. Kita T. Kada S. Yoshimoto M. Nakahata T.. (2008). Bone marrow-derived cells expressing Iba1 are constitutively present as resident tissue macrophages in the mouse cochlea. J. Neurosci. Res. 86, 1758–1767. 10.1002/jnr.21625 18253944

[B121] O’Sullivan S. A. Gasparini F. Mir A. K. Dev K. K. (2016). Fractalkine shedding is mediated by p38 and the ADAM10 protease under pro-inflammatory conditions in human astrocytes. J. Neuroinflammation 13:189. 10.1186/s12974-016-0659-7 27549131 PMC4994207

[B122] Oved J. H. Barrett D. M. Teachey D. T. (2019). Cellular therapy: immune-related complications. Immunol. Rev. 290, 114–126. 10.1111/imr.12768 31355491 PMC7041800

[B123] Pabon M. M. Bachstetter A. D. Hudson C. E. Gemma C. Bickford P. C. (2011). CX3CL1 reduces neurotoxicity and microglial activation in a rat model of Parkinson’s disease. J. Neuroinflammation 8:9. 10.1186/1742-2094-8-9 21266082 PMC3039584

[B124] Palmada M. Centelles J. J. (1998). Excitatory amino acid neurotransmission. Pathways for metabolism, storage and reuptake of glutamate in brain. Front. Biosci. 3, d701–d718. 10.2741/a314 9665875

[B125] Pan Y. Lloyd C. Zhou H. Dolich S. Deeds J. Gonzalo J. A.. (1997). Neurotactin, a membrane-anchored chemokine upregulated in brain inflammation. Nature 387, 611–617. 10.1038/42491 9177350

[B126] Paolicelli R. C. Bisht K. Tremblay M. E. (2014). Fractalkine regulation of microglial physiology and consequences on the brain and behavior. Front. Cell. Neurosci. 8:129. 10.3389/fncel.2014.00129 24860431 PMC4026677

[B127] Paolicelli R. C. Bolasco G. Pagani F. Maggi L. Scianni M. Panzanelli P.. (2011). Synaptic pruning by microglia is necessary for normal brain development. Science 333, 1456–1458. 10.1126/science.1202529 21778362

[B128] Peng B. Xiao J. Wang K. So K. F. Tipoe G. L. Lin B. (2014). Suppression of microglial activation is neuroprotective in a mouse model of human retinitis pigmentosa. J. Neurosci. 34, 8139–8150. 10.1523/JNEUROSCI.5200-13.2014 24920619 PMC6608244

[B129] Polymeropoulos M. H. Lavedan C. Leroy E. Ide S. E. Dehejia A. Dutra A.. (1997). Mutation in the alpha-synuclein gene identified in families with Parkinson’s disease. Science 276, 2045–2047. 10.1126/science.276.5321.2045 9197268

[B130] Ransohoff R. M. (2009). Chemokines and chemokine receptors: standing at the crossroads of immunobiology and neurobiology. Immunity 31, 711–721. 10.1016/j.immuni.2009.09.010 19836265 PMC2787682

[B131] Ravichandran K. S. (2010). Find-me and eat-me signals in apoptotic cell clearance: progress and conundrums. J. Exp. Med. 207, 1807–1817. 10.1084/jem.20101157 20805564 PMC2931173

[B132] Ravichandran K. S. (2011). Beginnings of a good apoptotic meal: the find-me and eat-me signaling pathways. Immunity 35, 445–455. 10.1016/j.immuni.2011.09.004 22035837 PMC3241945

[B133] Ridderstad Wollberg A. Ericsson-Dahlstrand A. Jureus A. Ekerot P. Simon S. Nilsson M.. (2014). Pharmacological inhibition of the chemokine receptor CX3CR1 attenuates disease in a chronic-relapsing rat model for multiple sclerosis. Proc. Natl. Acad. Sci. U S A 111, 5409–5414. 10.1073/pnas.1316510111 24706865 PMC3986185

[B134] Roseti C. Fucile S. Lauro C. Martinello K. Bertollini C. Esposito V.. (2013). Fractalkine/CX3CL1 modulates GABAA currents in human temporal lobe epilepsy. Epilepsia 54, 1834–1844. 10.1111/epi.12354 24032743

[B135] Ross O. A. Braithwaite A. T. Skipper L. M. Kachergus J. Hulihan M. M. Middleton F. A.. (2008). Genomic investigation of alpha-synuclein multiplication and parkinsonism. Ann. Neurol. 63, 743–750. 10.1002/ana.21380 18571778 PMC3850281

[B136] Roy I. Evans D. B. Dwinell M. B. (2014). Chemokines and chemokine receptors: update on utility and challenges for the clinician. Surgery 155, 961–973. 10.1016/j.surg.2014.02.006 24856117 PMC4390364

[B137] Salvi V. Sozio F. Sozzani S. Del Prete A. (2017). Role of atypical chemokine receptors in microglial activation and polarization. Front. Aging Neurosci. 9:148. 10.3389/fnagi.2017.00148 28603493 PMC5445112

[B138] Sato E. Shick H. E. Ransohoff R. M. Hirose K. (2008). Repopulation of cochlear macrophages in murine hematopoietic progenitor cell chimeras: the role of CX3CR1. J. Comp. Neurol. 506, 930–942. 10.1002/cne.21583 18085589

[B139] Sato E. Shick H. E. Ransohoff R. M. Hirose K. (2010). Expression of fractalkine receptor CX3CR1 on cochlear macrophages influences survival of hair cells following ototoxic injury. J. Assoc. Res. Otolaryngol. 11, 223–234. 10.1007/s10162-009-0198-3 19936834 PMC2862920

[B140] Schafer D. P. Stevens B. (2015). Microglia function in central nervous system development and plasticity. Cold Spring Harb. Perspect Biol. 7:a020545. 10.1101/cshperspect.a020545 26187728 PMC4588063

[B141] Schulz C. Schafer A. Stolla M. Kerstan S. Lorenz M. von Bruhl M. L.. (2007). Chemokine fractalkine mediates leukocyte recruitment to inflammatory endothelial cells in flowing whole blood: a critical role for P-selectin expressed on activated platelets. Circulation 116, 764–773. 10.1161/CIRCULATIONAHA.107.695189 17679613

[B142] Sharma A. K. Reams R. Y. Jordan W. H. Miller M. A. Thacker H. L. Snyder P. W. (2007). Mesial temporal lobe epilepsy: pathogenesis, induced rodent models and lesions. Toxicol. Pathol. 35, 984–999. 10.1080/01926230701748305 18098044

[B143] Sheldon A. L. Robinson M. B. (2007). The role of glutamate transporters in neurodegenerative diseases and potential opportunities for intervention. Neurochem. Int. 51, 333–355. 10.1016/j.neuint.2007.03.012 17517448 PMC2075474

[B144] Sheridan G. K. Murphy K. J. (2013). Neuron-glia crosstalk in health and disease: fractalkine and CX3CR1 take centre stage. Open Biol. 3:130181. 10.1098/rsob.130181 24352739 PMC3877844

[B145] Sheridan G. K. Wdowicz A. Pickering M. Watters O. Halley P. O’Sullivan N. C.. (2014). CX3CL1 is up-regulated in the rat hippocampus during memory-associated synaptic plasticity. Front. Cell. Neurosci. 8:233. 10.3389/fncel.2014.00233 25161610 PMC4130185

[B146] Shi X. (2010). Resident macrophages in the cochlear blood-labyrinth barrier and their renewal via migration of bone-marrow-derived cells. Cell Tissue Res. 342, 21–30. 10.1007/s00441-010-1040-2 20838812

[B147] Stankovic K. Rio C. Xia A. Sugawara M. Adams J. C. Liberman M. C.. (2004). Survival of adult spiral ganglion neurons requires erbB receptor signaling in the inner ear. J. Neurosci. 24, 8651–8661. 10.1523/JNEUROSCI.0733-04.2004 15470130 PMC6729966

[B148] Stephenson J. Nutma E. van der Valk P. Amor S. (2018). Inflammation in CNS neurodegenerative diseases. Immunology 154, 204–219. 10.1111/imm.12922 29513402 PMC5980185

[B149] Stojkovic L. Djuric T. Stankovic A. Dincic E. Stancic O. Veljkovic N.. (2012). The association of V249I and T280M fractalkine receptor haplotypes with disease course of multiple sclerosis. J. Neuroimmunol. 245, 87–92. 10.1016/j.jneuroim.2011.12.028 22261545

[B150] Sun S. Yu H. Yu H. Honglin M. Ni W. Zhang Y.. (2015). Inhibition of the activation and recruitment of microglia-like cells protects against neomycin-induced ototoxicity. Mol. Neurobiol. 51, 252–267. 10.1007/s12035-014-8712-y 24781382

[B151] Tan W. J. Thorne P. R. Vlajkovic S. M. (2016). Characterisation of cochlear inflammation in mice following acute and chronic noise exposure. Histochem. Cell Biol. 146, 219–230. 10.1007/s00418-016-1436-5 27109494

[B152] Tang J. Kern T. S. (2011). Inflammation in diabetic retinopathy. Prog. Retin. Eye Res. 30, 343–358. 10.1016/j.preteyeres.2011.05.002 21635964 PMC3433044

[B153] Tauber A. I. (2003). Metchnikoff and the phagocytosis theory. Nat. Rev. Mol. Cell Biol. 4, 897–901. 10.1038/nrm1244 14625539

[B154] Thome A. D. Standaert D. G. Harms A. S. (2015). Fractalkine signaling regulates the inflammatory response in an alpha-synuclein model of parkinson disease. PLoS One 10:e0140566. 10.1371/journal.pone.0140566 26469270 PMC4607155

[B155] Tong L. Strong M. K. Kaur T. Juiz J. M. Oesterle E. C. Hume C.. (2015). Selective deletion of cochlear hair cells causes rapid age-dependent changes in spiral ganglion and cochlear nucleus neurons. J. Neurosci. 35, 7878–7891. 10.1523/JNEUROSCI.2179-14.2015 25995473 PMC4438131

[B156] Tuo J. Smith B. C. Bojanowski C. M. Meleth A. D. Gery I. Csaky K. G.. (2004). The involvement of sequence variation and expression of CX3CR1 in the pathogenesis of age-related macular degeneration. FASEB J. 18, 1297–1299. 10.1096/fj.04-1862fje 15208270 PMC1971128

[B157] Ueno M. Fujita Y. Tanaka T. Nakamura Y. Kikuta J. Ishii M.. (2013). Layer V cortical neurons require microglial support for survival during postnatal development. Nat. Neurosci. 16, 543–551. 10.1038/nn.3358 23525041

[B158] Umehara H. Bloom E. T. Okazaki T. Nagano Y. Yoshie O. Imai T. (2004). Fractalkine in vascular biology: from basic research to clinical disease. Arterioscler. Thromb. Vasc. Biol. 24, 34–40. 10.1161/01.ATV.0000095360.62479.1F 12969992

[B159] Wang K. Peng B. Lin B. (2014). Fractalkine receptor regulates microglial neurotoxicity in an experimental mouse glaucoma model. Glia 62, 1943–1954. 10.1002/glia.22715 24989686

[B160] Wang S. K. Xue Y. Rana P. Hong C. M. Cepko C. L. (2019). Soluble CX3CL1 gene therapy improves cone survival and function in mouse models of retinitis pigmentosa. Proc. Natl. Acad. Sci. U S A 116, 10140–10149. 10.1073/pnas.1901787116 31036641 PMC6525490

[B161] Wang Y. Hirose K. Liberman M. C. (2002). Dynamics of noise-induced cellular injury and repair in the mouse cochlea. J. Assoc. Res. Otolaryngol. 3, 248–268. 10.1007/s101620020028 12382101 PMC3202415

[B162] White G. E. Greaves D. R. (2012). Fractalkine: a survivor’s guide: chemokines as antiapoptotic mediators. Arterioscler. Thromb. Vasc. Biol. 32, 589–594. 10.1161/ATVBAHA.111.237412 22247260

[B163] Wojdasiewicz P. Poniatowski L. A. Kotela A. Deszczynski J. Kotela I. Szukiewicz D. (2014). The chemokine CX3CL1 (fractalkine) and its receptor CX3CR1: occurrence and potential role in osteoarthritis. Arch. Immunol. Ther. Exp. (Warsz) 62, 395–403. 10.1007/s00005-014-0275-0 24556958 PMC4164853

[B164] Wood M. B. Zuo J. (2017). The contribution of immune infiltrates to ototoxicity and cochlear hair cell loss. Front. Cell. Neurosci. 11:106. 10.3389/fncel.2017.00106 28446866 PMC5388681

[B165] Xie D. He M. Hu X. (2019). Microglia/macrophage diversities in central nervous system physiology and pathology. CNS Neurosci. Ther. 25, 1287–1289. 10.1111/cns.13257 31793210 PMC7154592

[B166] Yang W. Vethanayagam R. R. Dong Y. Cai Q. Hu B. H. (2015). Activation of the antigen presentation function of mononuclear phagocyte populations associated with the basilar membrane of the cochlea after acoustic overstimulation. Neuroscience 303, 1–15. 10.1016/j.neuroscience.2015.05.081 26102003 PMC4532582

[B167] Zabel M. K. Zhao L. Zhang Y. Gonzalez S. R. Ma W. Wang X.. (2016). Microglial phagocytosis and activation underlying photoreceptor degeneration is regulated by CX3CL1-CX3CR1 signaling in a mouse model of retinitis pigmentosa. Glia 64, 1479–1491. 10.1002/glia.23016 27314452 PMC4958518

[B168] Zhan Y. Paolicelli R. C. Sforazzini F. Weinhard L. Bolasco G. Pagani F.. (2014). Deficient neuron-microglia signaling results in impaired functional brain connectivity and social behavior. Nat. Neurosci. 17, 400–406. 10.1038/nn.3641 24487234

[B169] Zhang W. Dai M. Fridberger A. Hassan A. Degagne J. Neng L.. (2012). Perivascular-resident macrophage-like melanocytes in the inner ear are essential for the integrity of the intrastrial fluid-blood barrier. Proc. Natl. Acad. Sci. U S A 109, 10388–10393. 10.1073/pnas.1205210109 22689949 PMC3387119

[B170] Zilberstein Y. Liberman M. C. Corfas G. (2012). Inner hair cells are not required for survival of spiral ganglion neurons in the adult cochlea. J. Neurosci. 32, 405–410. 10.1523/JNEUROSCI.4678-11.2012 22238076 PMC3678770

[B171] Zlotnik A. Yoshie O. (2000). Chemokines: a new classification system and their role in immunity. Immunity 12, 121–127. 10.1016/s1074-7613(00)80165-x 10714678

[B172] Zlotnik A. Yoshie O. Nomiyama H. (2006). The chemokine and chemokine receptor superfamilies and their molecular evolution. Genome Biol. 7:243. 10.1186/gb-2006-7-12-243 17201934 PMC1794421

